# Structural insights into Mitoguardin-1-mediated mitochondrial contact surfaces in fusion and lipid binding

**DOI:** 10.1038/s41421-026-00931-3

**Published:** 2026-10-29

**Authors:** Kanglong Yang, Jie Deng, Fan Yu, Lei Wang, Xiao Yuan, Ke Ruan, Xuebiao Yao, Yunyu Shi, Liang Zhang

**Affiliations:** Center for Advanced Interdisciplinary Science and Biomedicine of IHM; Ministry of Education Key Laboratory for Membraneless Organelles and Cellular Dynamics; Hefei National Research Center for Cross-disciplinary Science; Division of Life Sciences and Medicine, University of Science and Technology of China, Hefei, China

**Keywords:** Mitochondria, X-ray crystallography

Dear Editor,

Mitochondrial fusion is essential for organelle homeostasis^1,2^ and is regulated by MFN1/2, Mito-PLD, and the Mitoguardin (MIGA) family^3,4^. MIGA proteins localize to the outer mitochondrial membrane^5^, where they have been implicated in mitochondrial fusion and lipid homeostasis through interactions with Mito-PLD and membrane contact sites^6,7^. In particular, MIGA2 promotes triglyceride synthesis at mitochondria-associated membrane contact sites^8^, whereas MIGA1/2 deficiency causes defects in lipid metabolism in vivo^9^. Human MIGA1 contains four conserved domains^10^, an N-terminal TM, a coiled-coil domain, an FFAT motif, and a C-terminal LD-targeting domain (Supplementary Fig. S1a)^11,12^. However, despite its established roles in mitochondrial fusion and lipid homeostasis^13,14^, the structural basis of MIGA1 oligomerization, the molecular determinants of lipid binding, and their relationship to mitochondrial fusion remain unknown.

To elucidate the molecular mechanisms of MIGA proteins, we first expressed the LD-targeting domains of human MIGA1 (hMIGA1) and MIGA2 (hMIGA2) in *Escherichia coli*. Gel filtration chromatography and static light scattering analysis showed that hMIGA1 exists as monomers and hexamers, whereas hMIGA2 displays a distinct oligomeric state (Supplementary Fig. S1b–d). MBP pull-down assays further demonstrated that both proteins form homo-oligomers and can also assemble into hetero-oligomers (Supplementary Fig. S2a, b). We next determined the 2.6 Å crystal structure of the hexameric hMIGA1 LD-targeting domain (Supplementary Table S1), revealing a hexamer composed of two stacked doughnut-shaped trimers (Fig. 1a, b). Within the upper trimer, residues N493, R494 and W495 insert into a pocket formed by the neighboring subunit, generating extensive hydrogen-bonding interactions with the neighboring subunit that stabilize the trimeric interface (Fig. 1c; Supplementary Fig. S2c, d). Mutation of these residues (N493A/R494A/W495A) disrupted hexamer formation, demonstrating that the trimeric interface is required for assembly of the hMIGA1 hexamer (Fig. 1d).

**Fig. 1:**
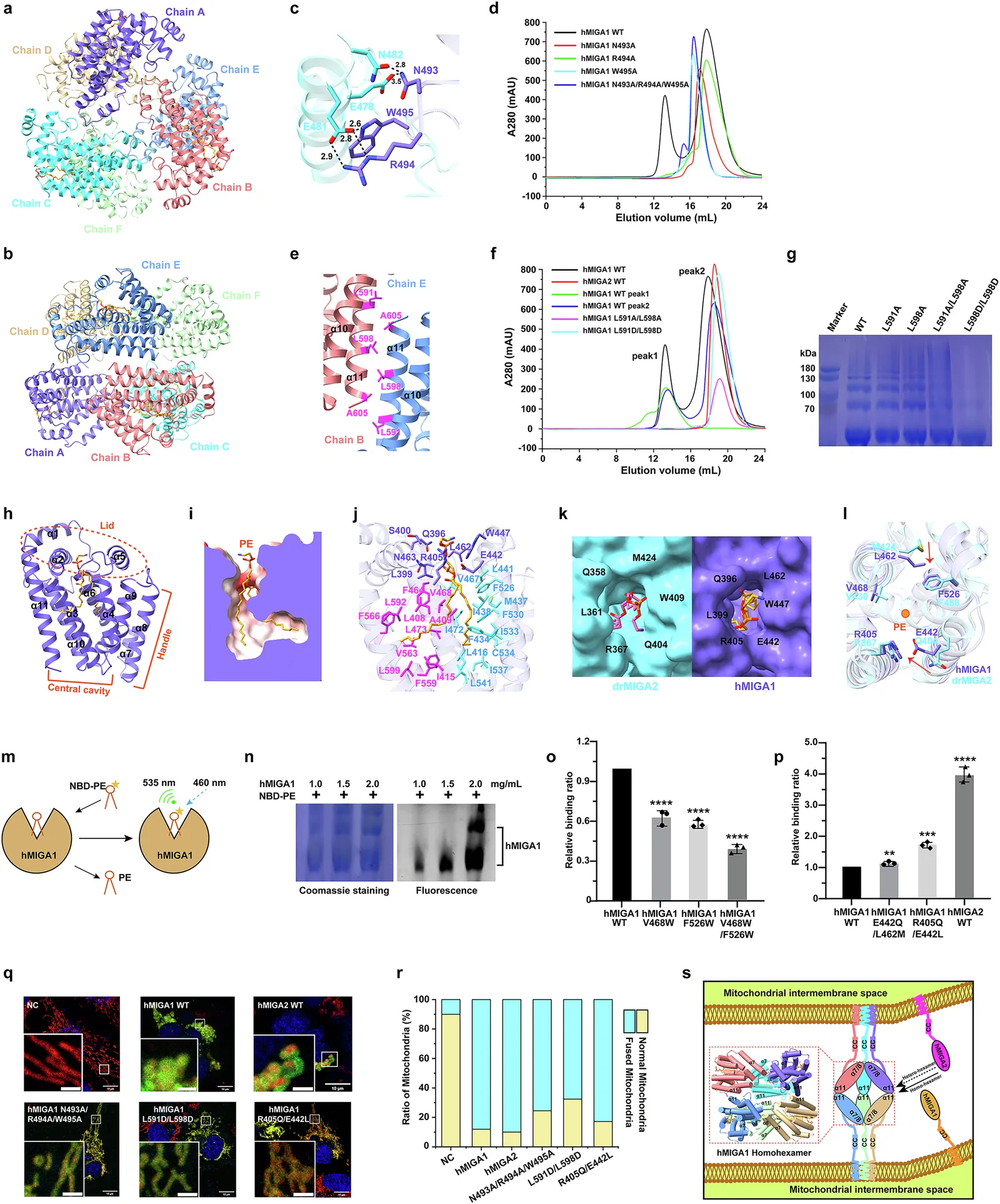
Structural insights into hMIGA1-mediated mitochondrial contact surfaces in fusion and lipid binding. **a**, **b** Overall structure of the hMIGA1 LD-targeting domain shown from different views. Individual subunits are colored separately. **c** Interaction interface within the upper-layer trimer. Black dashed lines indicate hydrogen bonds, with distances shown in Å. **d** Gel filtration profiles of wild-type and mutant hMIGA1 proteins. The peak eluting at ~12 mL corresponds to the apparent hexameric fraction (~220 kDa), whereas the peaks between ~16 and 20 mL correspond to the apparent monomeric fraction (~40 kDa). **e** Hydrophobic interface between two trimers mediated by the C-terminal α11 helices. **f** Gel filtration profiles of wild-type and mutant hMIGA1 and hMIGA2 proteins. Peak 1 and peak 2 correspond to the apparent hexameric and monomeric fractions, respectively. **g** BN-PAGE analysis of wild-type and mutant hMIGA1 proteins. **h** Cartoon representation of a single hMIGA1 subunit. **i** Cutaway surface view showing the central hydrophobic cavity of hMIGA1. **j** Structural view of phosphatidylethanolamine (PE) bound within the hMIGA1 lipid-binding pocket. Residues contacting the phospholipid head group and acyl chains are highlighted. **k** Comparison of the lipid-binding pockets of drMIGA2 (PDB: 7X15) and hMIGA1. **l** Comparison of key residues within the lipid-binding pockets of hMIGA1 and hMIGA2. Red arrows indicate residue movements. **m** Schematic illustration of the NBD-PE displacement assay. **n** BN-PAGE analysis of NBD-PE binding by purified hMIGA1 from SEC Peak 1. Left, Coomassie staining; right, fluorescence. Increasing protein concentrations were loaded from left to right. **o**, **p** Relative lipid-binding activity of wild-type and mutant hMIGA1 proteins. Data are presented as mean ± SD from three independent experiments. Statistical significance was determined using an unpaired two-tailed *t*-test (**P < 0.01, ***P < 0.001, ****P < 0.0001). **q** Representative confocal images showing mitochondrial morphology in cells expressing GFP-tagged wild-type or mutant hMIGA1 and hMIGA2 proteins. Nuclei were stained with Hoechst (blue), and mitochondria were labeled with MitoTracker (red). **r** Quantification of mitochondrial morphology in cells expressing wild-type or mutant hMIGA1 and hMIGA2 proteins. A total of more than 50 cells from three independent biological replicates were analyzed for each group. **s** Proposed model of hMIGA1-mediated mitochondrial fusion. Hexameric assembly of the hMIGA1 LD-targeting domain promotes recruitment of the mitochondrial fusion machinery and facilitates outer mitochondrial membrane fusion.

Two trimeric rings assemble into a cylindrical hexamer through antiparallel packing of the C-terminal α11 helices (Fig. 1e; Supplementary Fig. S2e). Hydrophobic interactions involving L591, L598, and A605 stabilize this interface (Supplementary Fig. S2f). To validate that the proposed interface is not a crystallographic artifact, DSS cross-linking was performed and further supported the hexameric interface (Supplementary Fig. S2g–h). Similar cross-linking patterns were observed for hMIGA2 and the hMIGA1-hMIGA2 complex (Supplementary Fig. S3a–d and Table S2), although intermolecular cross-links within hMIGA2 were not detected, suggesting differences in oligomerization between the two proteins.

These observations prompted us to investigate the functional significance of the hexamer interface. Purified hMIGA1 exists in equilibrium between hexameric and monomeric states (Fig. 1f). Mutation of the α11 interface residues L591 and L598 disrupted hexamer formation, as demonstrated by SEC, BN-PAGE and MBP pull-down assays (Fig. 1f, g; Supplementary Fig. S3e). To determine whether this oligomer also exists under physiological conditions, we performed DSS-mediated cross-linking in cells. Immunoblotting of endogenous MIGA1 revealed a prominent high-molecular-weight species consistent with the predicted size of the hexamer following DSS treatment (Supplementary Fig. S3f), providing direct evidence that full-length MIGA1 forms higher-order oligomers in cells. Together, these findings establish the hexamer as the physiologically relevant oligomeric state of MIGA1.

The hMIGA1 monomer adopts a cup-shaped fold composed of eleven α-helices surrounding a large hydrophobic cavity, a structural feature conserved among previously reported lipid-binding proteins (Fig. 1h; Supplementary Fig. S4a–d). Additional electron density within this cavity was consistent with a bound phospholipid, with the phosphate head group stabilized by R405 and the acyl chains accommodated within the hydrophobic pocket (Fig. 1i, j; Supplementary Fig. S4e–i). The lipid-binding residues are highly conserved, suggesting an evolutionarily conserved lipid-binding mechanism (Supplementary Fig. S5). Structural comparison with ceMIGA2^7^ and drMIGA2^15^ revealed a highly conserved overall fold but a smaller lipid-binding pocket in hMIGA1 (Fig. 1k; Supplementary Fig. S6a–e). This difference results primarily from inward movement of several pocket residues, including R405, E442 and L462, and may partially explain the weaker lipid-binding activity of hMIGA1 reported previously.

To explore whether divergent lipid‑binding capacities of human MIGA proteins are associated with lipid‑binding pocket alterations, we compared pocket sequences of drMIGA2, hMIGA2 and hMIGA1. Although pocket sequences are largely conserved, hMIGA1 shows distinct residues at R405, E442 and L462 relative to MIGA2 homologs, while lipid hydrocarbon‑chain‑interacting residues (V468, F526) are highly conserved (Supplementary Fig. S7a). Structural analysis reveals inward displacement of L462 and E442 side‑chains in hMIGA1 vs M424 and Q404 of drMIGA2, which shrinks the pocket volume (Fig. 1l). This inward shift enables R405‑E442 hydrogen bonding unique to hMIGA2‑absent hMIGA1. Further multi‑chain comparisons of hMIGA1 demonstrate conserved R405 for lipid head‑group recognition but variable E442 side‑chain conformation (Supplementary Fig. S7b). R405‑E442 hydrogen bonds form in chains A/C but disappear in B/E due to outward E442 displacement (Supplementary Fig. S7c), indicating dynamic R405‑E442 interactions. E442 conformational rearrangement may act as a molecular switch governing hMIGA1 lipid‑binding activity.

To evaluate the functional significance of the lipid-binding pocket, we performed NBD-PE displacement assays^15^ using wild-type and mutant hMIGA1 proteins (Fig. 1m, n). Mutations of conserved hydrophobic pocket residues (V468W, F526W or V468W/F526W) progressively reduced lipid-binding activity, demonstrating that the hydrophobic cavity is required for efficient lipid binding (Fig. 1o). Consistent with previous reports, hMIGA2 exhibited stronger lipid-binding activity than hMIGA1 (Fig. 1p). Moreover, mutation of the R405/E442 switch modestly enhanced lipid binding, supporting a regulatory role for this region in controlling pocket accessibility. We next investigated whether oligomerization contributes to lipid binding by comparing purified wild-type hMIGA1 in the hexameric and monomeric states together with oligomerization-defective mutants. Disruption of either the trimeric (N493A/R494A/W495A) or hexameric (L591D/L598D) interface reduced lipid-binding activity, unexpectedly, the isolated monomer retained activity comparable to that of the hexamer (Supplementary Fig. S8). These findings suggest that the integrity of the oligomerization interfaces, rather than oligomeric state alone, contributes to efficient lipid binding.

To determine whether hexamer integrity is required for mitochondrial fusion, GFP-tagged wild-type hMIGA1, hMIGA2, and oligomerization-deficient hMIGA1 mutants were expressed in COS7 cells. Wild-type hMIGA1 and hMIGA2 localized to mitochondria, consistent with their roles as outer mitochondrial membrane proteins (Fig. 1q; Supplementary Fig. S9a, b). In contrast, disruption of either the trimeric interface (N493A/R494A/W495A) or the hexameric interface (L591D/L598D) reduced the proportion of cells exhibiting hyperfused mitochondria compared with wild-type hMIGA1 (Fig. 1q; Supplementary Fig. S9c, d). Notably, the L591D/L598D mutant produced the strongest reduction in mitochondrial fusion (Fig. 1r), demonstrating that hexamer integrity is required for efficient hMIGA1-mediated mitochondrial fusion.

Having established that hexamer integrity is required for efficient mitochondrial fusion, we next investigated whether lipid-binding regulation also contributes to this process. We generated the R405Q/E442L mutant, which disrupts the lipid-binding switch and has previously been shown to enhance lipid binding in vitro. Overexpression of this mutant in COS7 cells reduced the proportion of cells exhibiting hyperfused mitochondria (Fig. 1q; Supplementary Fig. S9e, f). To further validate these findings and minimize potential artifacts associated with protein overexpression, endogenous hMIGA1 was depleted by siRNA and rescued with wild-type or mutant hMIGA1 constructs (Supplementary Fig. S10e). Together with the rescue experiments, these results demonstrate that both hexamer integrity and proper regulation of the lipid-binding pocket are required for efficient mitochondrial fusion mediated by hMIGA1 (Supplementary Fig. S10a–f). To investigate the mechanism underlying hMIGA1-mediated mitochondrial fusion, we examined mitochondrial recruitment of MFN1. Only the hexamer-disrupting mutant (L591D/L598D), but not the trimer-interface or lipid-binding mutants, impaired MFN1 recruitment (Supplementary Fig. S11), suggesting that hexamer integrity facilitates efficient recruitment of MFN1 to mitochondria.

Our findings provide the first structural characterization of the hMIGA1 LD-targeting domain, revealing a stable hexameric assembly required for efficient mitochondrial fusion. Unlike previous studies of MIGA2 that focused primarily on monomeric structure and lipid binding, our work links oligomerization, lipid-binding regulation, and mitochondrial fusion in hMIGA1 (Fig. 1s). These findings provide a structural framework for understanding how oligomerization and lipid-binding regulation coordinate mitochondrial membrane dynamics.

## Supplementary information

Supplementary Figs S1 to S11, Table S1

Supplementary Table S2

PDB validation
