# Supplementary material for: Structural insights into Mitoguardin-1-mediated mitochondrial contact surfaces in fusion and lipid binding
Source: Cell Discov. 2026 Oct 29;12:76. doi: 10.1038/s41421-026-00931-3 (PMC13720014; doi:10.1038/s41421-026-00931-3)
Supplement: Supplementary file 1 [file 41421_2026_931_MOESM1_ESM.pdf]

Structural insights into Mitoguardin-1-mediated mitochondrial contact surfaces in fusion and lipid binding

Kanglong Yang[1,#,*], Jie Deng[1,#], Fan Yu[1,#], Lei Wang[1], Xiao Yuan[1], Ke Ruan[1], Xuebiao Yao[1], Yunyu Shi[1,*], Liang Zhang[1,*]

[1] Center for Advanced Interdisciplinary Science and Biomedicine of IHM; Ministry of Education Key Laboratory for Membraneless Organelles and Cellular Dynamics; Hefei National Research Center for Cross-disciplinary Science; Division of Life Sciences and Medicine, University of Science and Technology of China, Hefei, Anhui, 230027, China;

[#] These authors contributed equally to this work

[*] To whom correspondence should be addressed. Liang Zhang, Tel: +86 551 63600441; Fax: +86 551 63601443; Email: zhangl99@ustc.edu.cn or Kanglong Yang, Email: klyang@mail.ustc.edu.cn

This Supplementary Materials file includes:

Materials and Methods
Figures S1 to S11
Table S1

Materials and Methods

Protein expression and purification

hMIGA1 (residues 350-620) and hMIGA2(residues 314-571) were amplified by PCR from the human brain cDNA library and cloned into the pET-28a (+) expression vector (Novagen), contained an N-terminal 6×His tag and a tobacco etch virus (TEV) protease cleavage site. All mutants of hMIGA1 were generated using a MutantBEST kit (Takara) and confirmed by DNA sequencing. All the proteins were expressed in Escherichia coli BL21 (DE3) cells (Novagen) cultured in LB medium at 37 °C to OD_{600} = 0.8, then shifted to 16 °C and induced with 0.5 mmol/L isopropyl β-D-1-thiogakactopyranoside (IPTG) for 24 hours. Bacterial pellets were resuspended in buffer A (20 mM Tris-HCl, 1 M NaCl, pH 7.5) and lysed by sonication on ice. Then, soluble proteins were purified with a Ni^{2+} -chelating column (Cytiva Life Sciences), followed by a Superdex 200 column (Cytiva Life Sciences) equilibrated with buffer B (20 mM Tris-HCl, 200 mM NaCl, pH 7.5). After being cleaved by TEV protease overnight at 4 °C to remove the 6×His tag, the purified protein was concentrated to ∼10 mg/mL in buffer B and stored at -80 °C. We also constructed MBP-hMIGA1 and MBP-hMIGA2 expression vectors and expressed these proteins in the same way for subsequent MBP pull-down assays.

MBP pull-down assays

The MBP alone control, MBP-tagged proteins (hMIGA1 and hMIGA2) were bound to BeyoMag™ Anti-MBP Magnetic Beads (Beyotime) in a buffer of 20 mM Tris, 200 mM NaCl, and 1 mM DTT at pH 7.5, and incubated at 4 °C for 4 hours. The beads were then spined down and washed three times with the washing buffer. Then the beads were incubated with recombinant hMIGA1 and hMIGA2 in a buffer including 20 mM Tris, 200 mM NaCl and 1 mM DTT at pH 7.5 for 6 hours at 4 °C. The beads were subsequently pelleted and washed three times using the same buffer. The proteins captured by beads were eluted and analyzed by SDS-PAGE.

Size exclusion chromatography with multi-angle light scattering (SEC-MALS)

A DAWN HELEOS-II (Wyatt Technology) multi-angle bright scattering detector and an Optilab T-rEX differential refractometer (Wyatt Technology) were used inline with a Superdex 200 Increase 10/300 GL (Cat# 29148721, Cytiva Life Science) column. The samples were run at a flow rate of 0.4 ml/min in the SEC-MALS buffer at room temperature. The data were analyzed using ASTRA 6.1 software.

Crystallization, data collection and structure determination

hMIGA1 was crystallized in 0.05 M potassium chloride, 1.1 M ammonium sulfate, 0.05 M Bis-tris (pH 7.0) at 16 °C by vapor diffusion in sitting drops. Crystals were soaked in cryo-protectant made of mother liquor supplemented with 25% glycerol before being flash-frozen in liquid nitrogen. Data sets were collected on Beamline 19U at Shanghai Synchrotron Radiation Facility (SSRF). The

structure of hMIGA1 was solved by molecular replacement with the program MOLREP, using AlphaFold structure as the search model. The structure of the hMIGA1 was refined by PHENIX.refine. Experimental structure factors and the coordinates of the final model have been deposited into the Protein Data Bank (PDB) with access code 9JCW. Crystal diffraction data and refinement statistics are summarized in Table S1.

Chemical cross-linking mass spectrometry

Dissolve an appropriate amount of DSS crosslinker in DMSO to prepare a 10 mM stock solution for subsequent use. Introduce the crosslinker into the diluted protein solution, ensuring that the final concentration of protein is 2 mg/mL and that of the crosslinker is 3 mM. The crosslinking reaction buffer consists of 20 mM HEPES, 200 mM NaCl, pH 7.5, mix thoroughly by pipetting and incubate at room temperature for one hour. Upon completion of the reaction, add Tris-HCl to achieve a final concentration of 50 mM (pH 8.0) in the crosslinking system and allow it to stand at room temperature for an additional 15 minutes to terminate the reaction. After completing the crosslinking process, centrifuge the sample and load it onto an SDS-PAGE gel for analysis of protein crosslinks. Once electrophoresis is complete, excise the gel strip containing crosslinked proteins and dissolve it for mass spectrometry analysis.

Lipid transport assay

For the lipid transport assays, 19 μl of 0.5 mg/ml wild-type hMIGA1 or one of its mutants and hMIGA2 in buffer B was mixed with 1 μl of 1 mg/ml NBD-PE (1,2-dioleoyl-sn-glycero-3-phosphoethanolamine-N-(7-nitro-2-1,3-benzoxadiazol-4-yl), Meryer) on ice. After 2 h, reaction products were subjected to BN-PAGE. The reaction products were detected by fluorescence (Image Quant LAS 4000, GE Healthcare) followed by Coomassie blue staining. Signal intensities were quantified with ImageJ software, and data were analyzed with GraphPad Prism 7.

Cell culture

Cell line COS7 are maintained in Dulbecco Modified Eagle Medium (DMEM) supplemented with 10% FBS, 100 units/mL penicillin, and 100 μg/mL streptomycin in a humidified atmosphere of 5% of CO2 in air at 37 °C. Cell medium was replaced every 2 days and the cells were sub-cultured once they reached 90% confluence.

Plasmid construction and cell transfection

To generate hMIGA1-GFP and hMIGA2-GFP expression vectors, cDNA encoding the full-length hMIGA1 and hMIGA2 genes were cloned and inserted into pcDNA3.1-eGFP plasmid, resulting in the expression of proteins fused to a C-terminal GFP tag. All mutants of hMIGA1 were generated using a MutanBEST kit (TaKaRa) and confirmed by DNA sequencing. Wild-type or mutant hMIGA1 and hMIGA2 expression vectors were used to transfect exponentially grown COS7 cells. Cells were transfected with 2.5 μg of GFP-hMIGA1 and hMIGA2 (WT or mutants) plasmids using

Hieff Trans® Universal Transfection Reagent (Yeasen).

Immunofluorescence staining and colocalization analysis

To investigate the subcellular localization of MFN1, immunofluorescence staining was performed. Cells grown on glass coverslips were incubated with MitoTracker Deep Red (Thermo Fisher Scientific) for 15 min at 37°C to label mitochondria. Cells were then fixed with 4% paraformaldehyde for 15 min, permeabilized with 0.2% Triton X-100 for 10 min, and blocked with 5% bovine serum albumin (BSA) for 1 h at room temperature.

The cells were incubated overnight at 4°C with mouse anti-MFN1 antibody (Proteintech, Cat. No. 66776-1-Ig, 1:1000) and rabbit anti-MIGA1 antibody (Invitrogen, Cat. No. PA5-53611, 1:1000). After washing three times with PBS, the cells were incubated for 1 h at room temperature with Alexa Fluor 488-conjugated goat anti-mouse IgG (to detect MFN1) and Alexa Fluor 594-conjugated goat anti-rabbit IgG (to detect MIGA1). Nuclei were counterstained with Hoechst 33342.

Fluorescence images were acquired using a STELLARIS STED confocal microscope (Leica Microsystems). Colocalization between MFN1 and mitochondria was quantified by calculating Manders' split coefficients (M1 and M2) using the Coloc2 plugin in Fiji/ImageJ.

Fluorescence colocalization

After transfecting 48 hours, COS7 cells were replanted in confocal dishes maintained in cell medium. Hoechst (Sigma) and Mito-tracker deep red (Thermo Fisher) were used for nucleus and mitochondrial staining for 15 mins. After washing three times with PBS, the cells were analyzed by a STELLARIS STED confocal system (Leica).

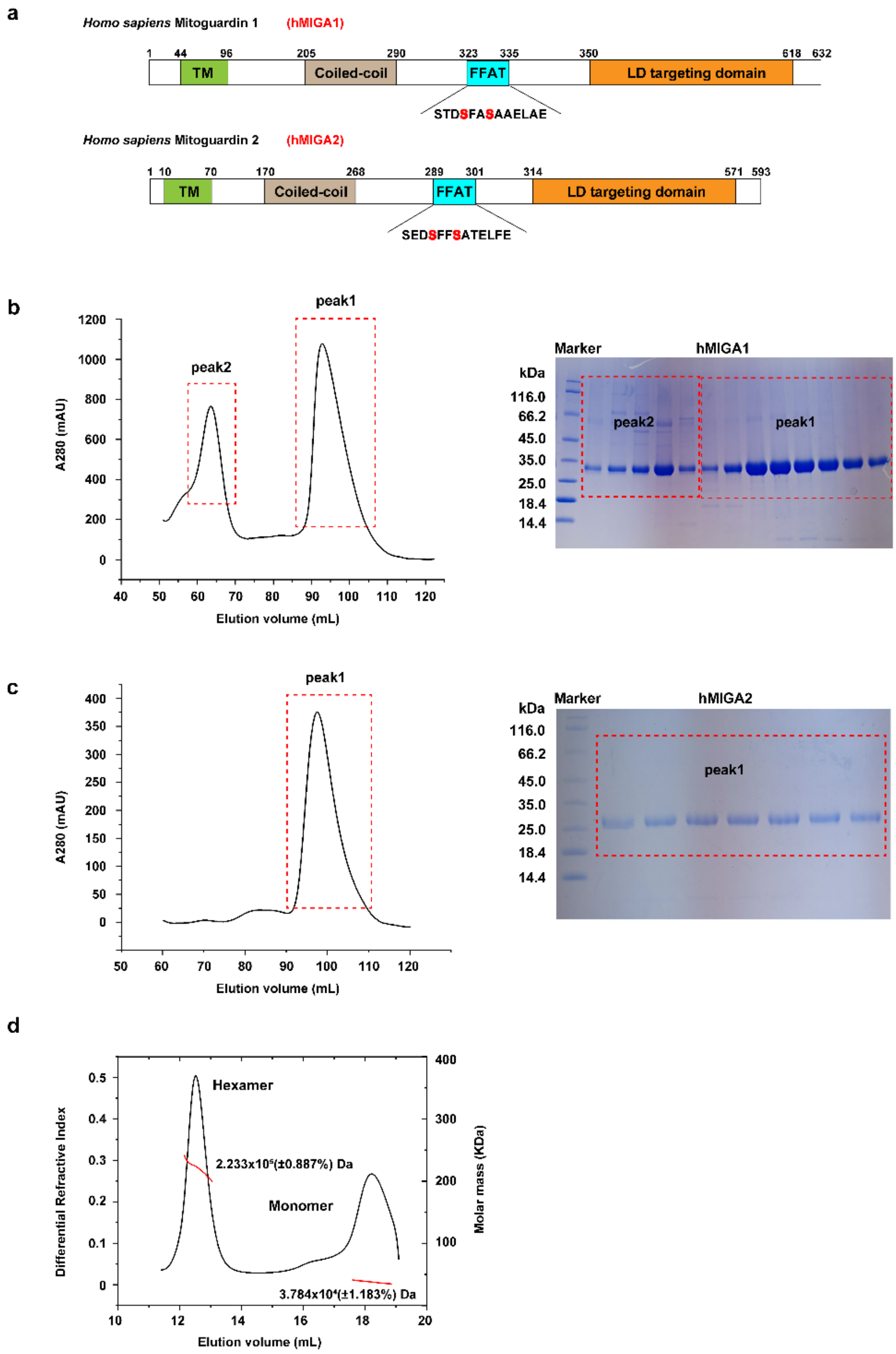

Supplementary Figure S1:

a Diagram illustrating the domain structure of hMIGA1 and hMIGA2. From the N-terminus, both proteins have a transmembrane domain (TM), coiled-coil domain, FFAT motif, and LD targeting domain. **b,c** Following gel filtration chromatography, the elution profiles of His-hMIGA1**(b)** and His-hMIGA2**(c)** proteins are presented. The red dashed box delineates the elution position of the target proteins along with the corresponding bands observed in the SDS-PAGE analysis. The peak exhibits a uniform and symmetrical shape, indicating a high level of protein purity. **d** SEC-MALS results for the hMIGA1 protein. The theoretical molecular weight of the LD-targeting domain of hMIGA1 is estimated to be approximately 34.6 kDa. The static light scattering experiments reveal two peaks corresponding to molecular weights of 223.3 kDa and 37.8 kDa, with the lower molecular weight peak representing the monomeric form and the higher molecular weight peak indicating the hexamer form.

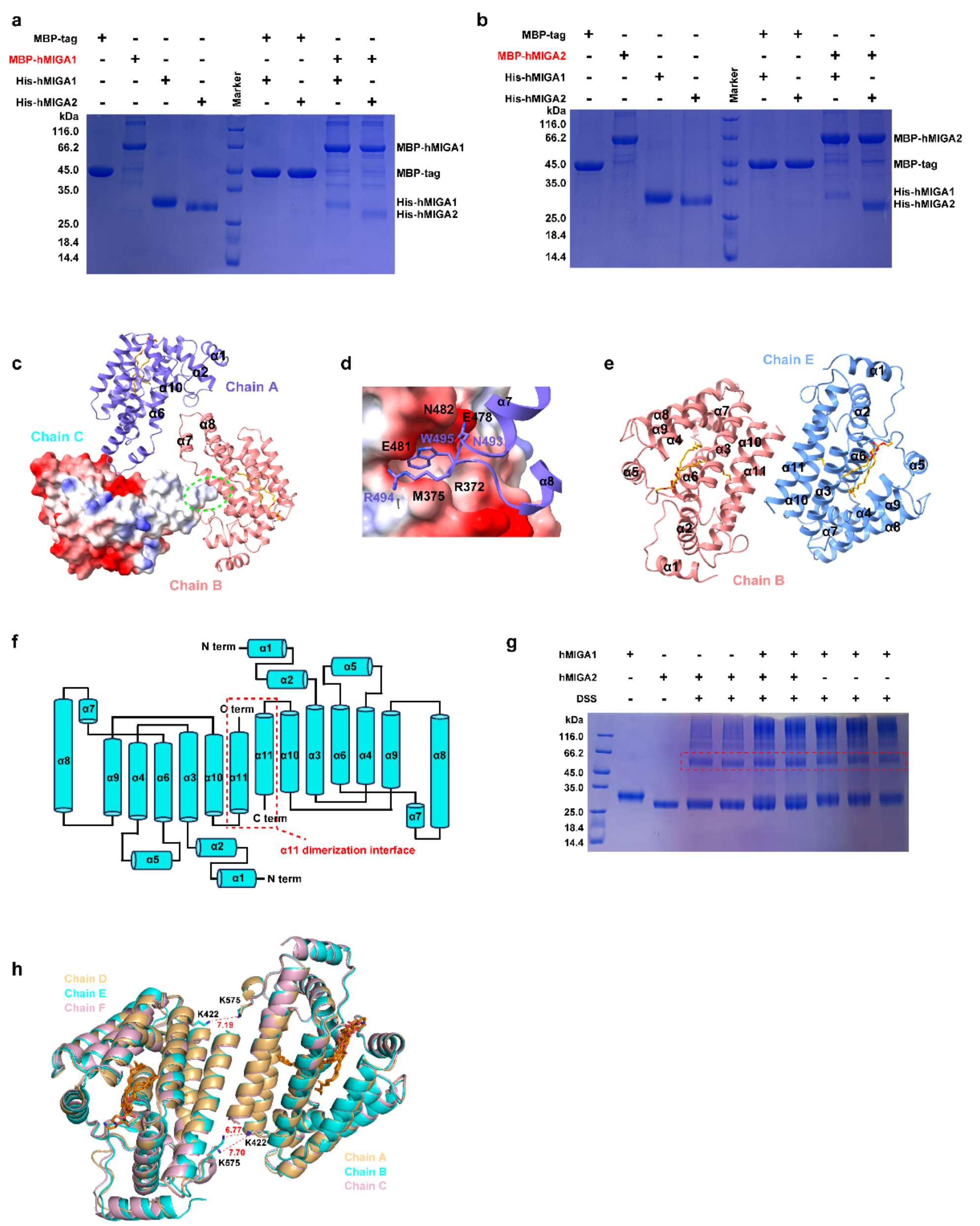

Supplementary Figure S2:

a,b In the MBP pull-down assay, hMIGA1 is capable of interacting with both hMIGA1 and hMIGA2, while hMIGA2 can similarly interact with both proteins. The right side of the figure presents the corresponding protein names for the bands marked in black. **c,d** Details of the interaction interface of the hMIGA1 upper layer trimer. **e** Dimeric interface by interdigitating through hydrophobic interactions involving the α11-helix at their C-terminus. **f** Schematic representation of the topological structure of the hMIGA1 protein, consisting of 11 α-helices, with the dimeric interface delineated by red dashed lines. **g** Cross-linked samples were prepared using the DSS cross-linking reagent and subsequently separated via SDS-PAGE, with dimeric protein samples outlined by red dashed lines. **h** Results from cross-linking mass spectrometry are overlaid onto the structure of the hMIGA1 protein, with the overall protein illustrated in cartoon representation and relevant residues depicted in stick format. Dashed lines and numbers represent the distances between the two residues.

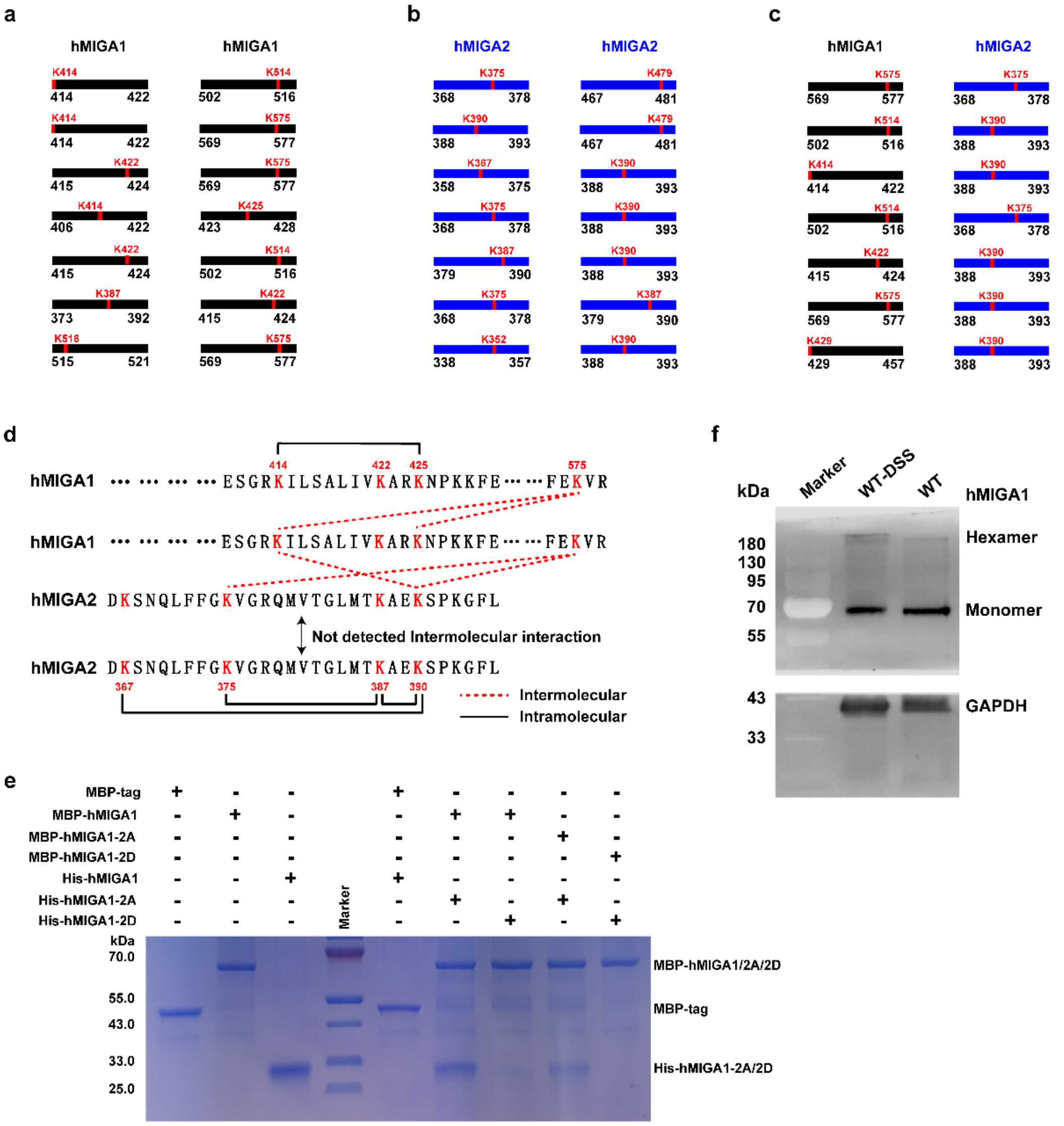

Supplementary Figure S3:

a-c A summary figure of the peptide segments from chemical cross-linking mass spectrometry for hMIGA1 and hMIGA2 is presented, with hMIGA1 peptide segments depicted in black, hMIGA2 peptide segments in blue, and cross-linked residues highlighted in red. **d** Cross-linking mass spectrometry results for hMIGA1 and hMIGA2 are illustrated, with key lysines involved in cross-linking highlighted in red. Numbers denote the positions of these lysines within their respective proteins. **e** MBP-pulldown experiments involving wild-type and mutant hMIGA1 proteins are illustrated, with the corresponding protein names indicated in black on the right side of the figure. **f** Western blot analysis of hMIGA1 oligomerization with or without DSS cross-linking in COS7 cells. Immunoblot was probed with anti-MIGA1 antibody; GAPDH served as loading control.

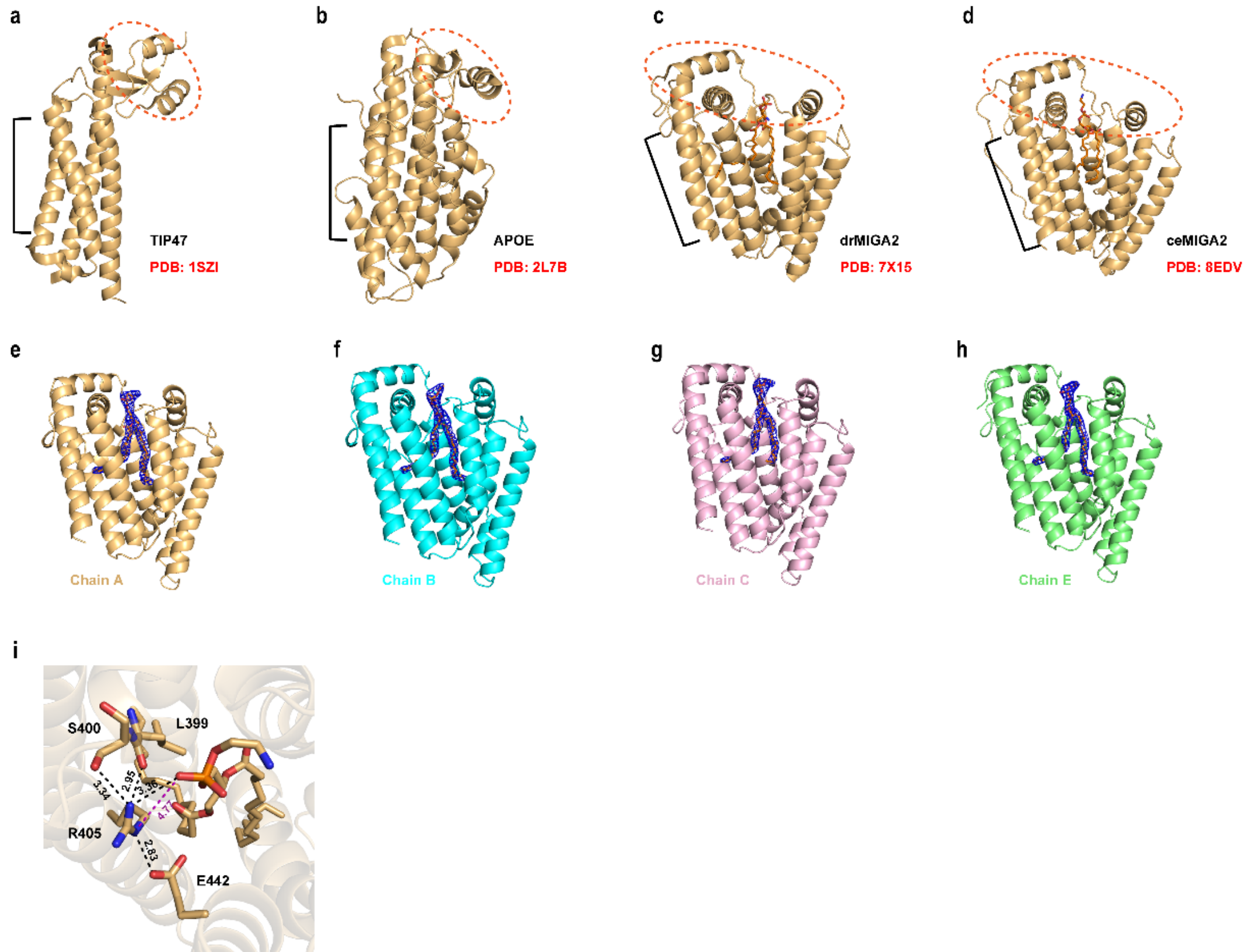

Supplementary Figure S4:

a-d Structural comparison of hMIGA1 with other lipid-binding proteins is provided, with the lid highlighted by dashed lines and the helix bundle extending to the left. The protein names and corresponding PDB IDs are indicated in black and red, respectively. **e-h** Distinct chains of the LD-targeting domain of hMIGA1 are illustrated in different colors using a cartoon representation, with lipids depicted in orange stick format. Blue grid illustrates the electron density of lipid molecules associated with the various chains (σ = 1). **i** Structural basis for the interaction of hMIGA1 proteins with the head of lipid molecules is presented. The overall hMIGA1 protein is illustrated in a transparent orange cartoon model, with key residues represented in stick format. Black dashed lines denote hydrogen bonds formed between residues, while magenta dashed lines indicate electrostatic interactions, with numbers representing distances.

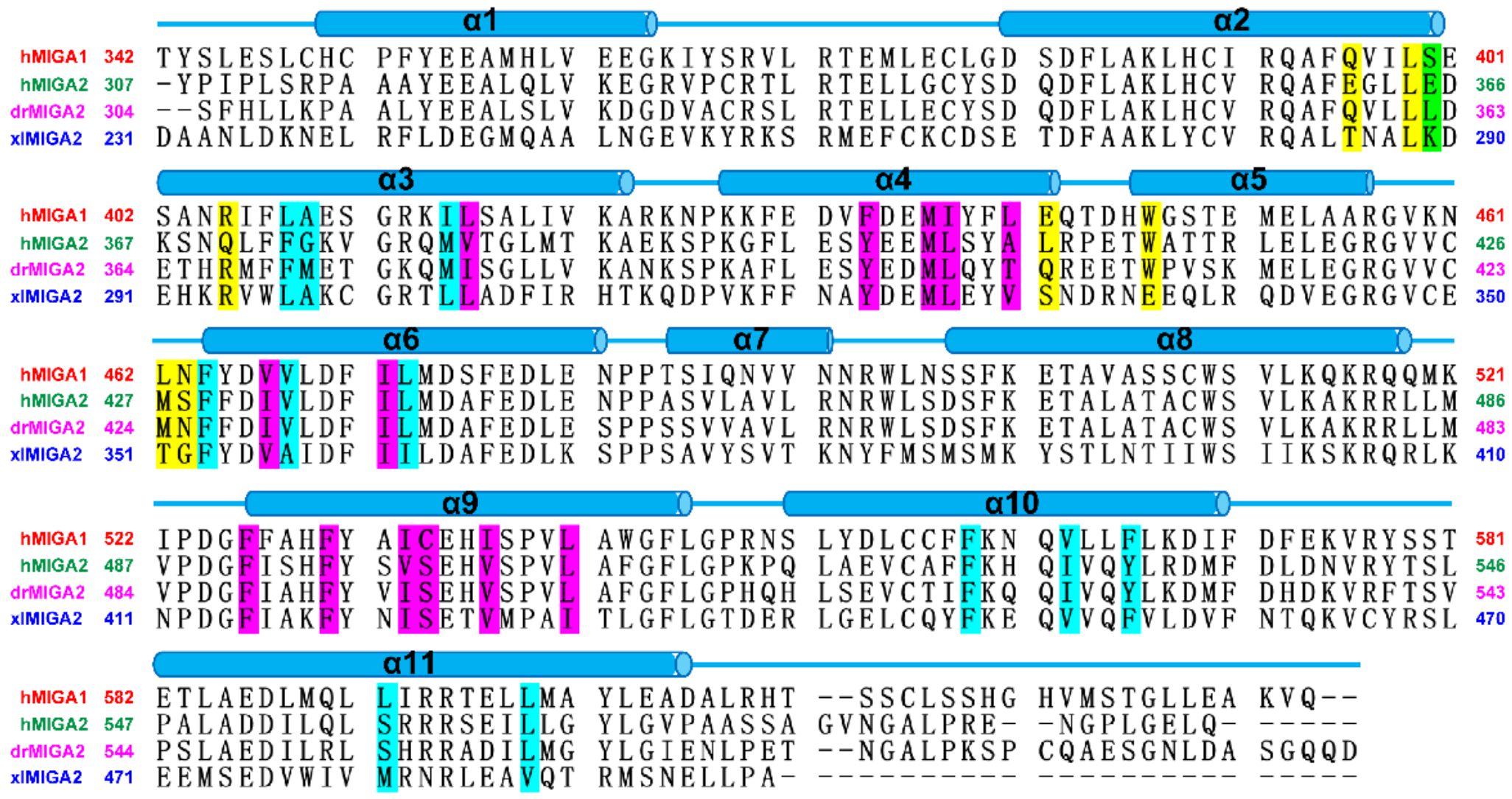

Supplementary Figure S5:

Sequence alignment of conservation of MIGA LD targeting domains. The secondary structure elements, derived from the crystal structure, are displayed above the sequences, while α helices are represented as cylinders, while loops are depicted as blue solid lines. The sequence alignment of the MIGA1 LD targeting domain from *Homo sapiens* (UniProt entry: Q8NAN2) and the sequence alignment of the MIGA2 LD targeting domain from *Homo sapiens* (UniProt entry: Q7L4E1), *Danio rerio* (UniProt entry: Q5BLE2), and *Xenopus laevis* (UniProt entry: Q6GR21) is also presented. The residues associated with the head and tail of the two hydrocarbon chains of PE, are illustrated in stick representation, utilizing yellow, magenta, and cyan to represent each component.

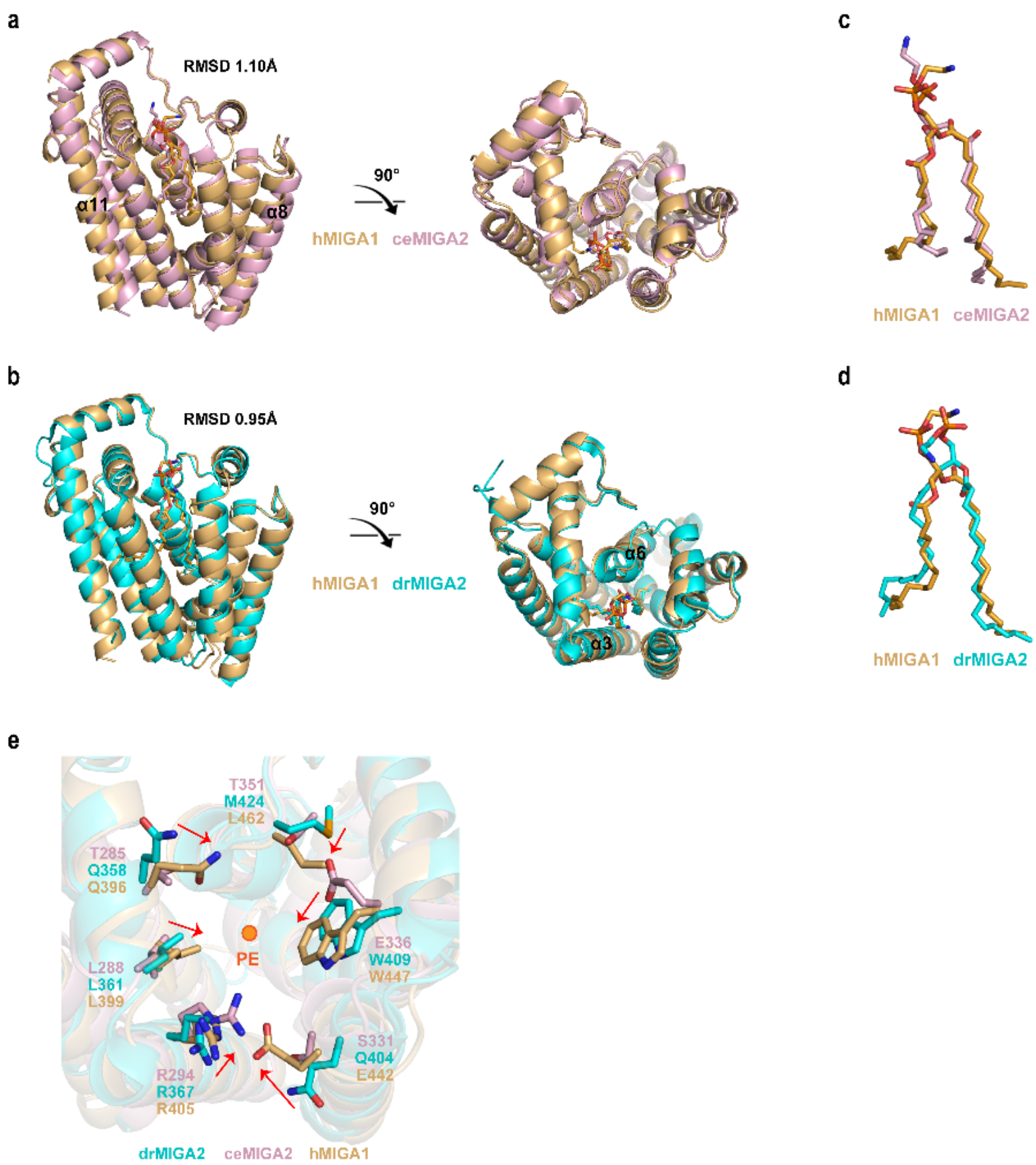

Supplementary Figure S6:

a-d Structural comparisons of the LD-targeting domains of hMIGA1 and ceMIGA2 **(a)**, along with drMIGA2 **(b)**, as well as structural comparisons of lipid binding in hMIGA1, ceMIGA2 **(c)** and drMIGA2 **(d)**. The proteins are depicted in a cartoon representation, while lipid molecules are illustrated in stick format. **e** Comparison of the lipid-binding pockets of hMIGA1, ceMIGA2 and drMIGA2 proteins, with proteins represented as transparent light orange and cyan cartoon models, respectively. Key residues are depicted in corresponding stick formats, with red arrows indicating the direction of residue shifts.

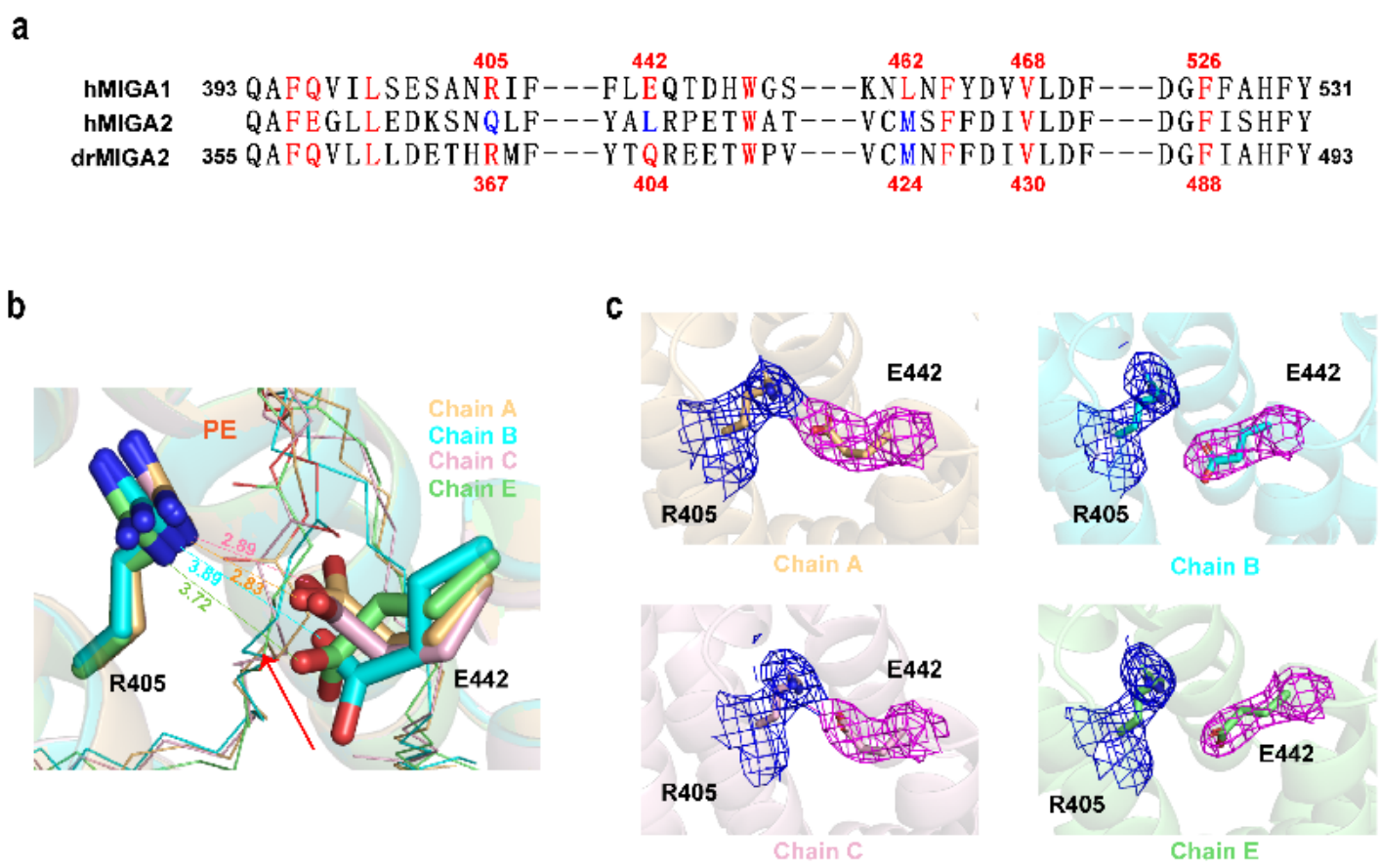

Supplementary Figure S7:

a Comparison of the amino acid sequences within the lipid-binding pocket of MIGA proteins, with red indicating relatively conserved residues and blue highlighting variable residues. **b** The "switch" mechanism within the lipid-binding pocket of different chains of hMIGA1, with R405 and E442 represented in stick format, and PE depicted in line representation. Dashed lines and numbers denote the distances between residues. **c** Residues R405 and E442 across the four chains exhibit a strong correlation with the electron density. The overall protein is depicted in a transparent cartoon model, with residues illustrated in stick representation. Grids of various colors represent the electron cloud density of the amino acids (σ = 1).

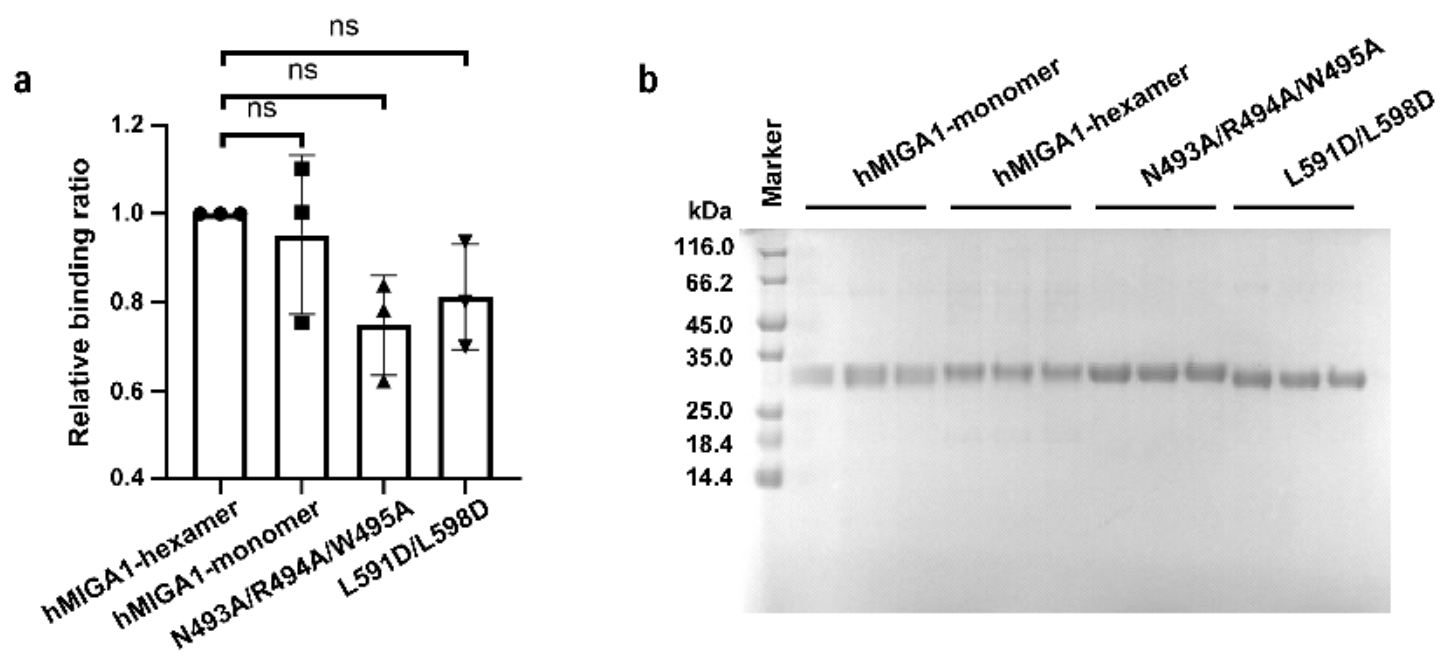

Supplementary Figure S8:

a Relative lipid-binding activity of hexameric hMIGA1, monomeric hMIGA1, and the hMIGA1 N493A/R494A/W495A and L591D/L598D mutants. Data are presented as mean ± SD from three independent biological replicates. The lipid-binding activity of hexameric hMIGA1 was normalized to 1. **b** Representative SDS-PAGE analysis showing comparable protein purity and loading for all hMIGA1 variants used in the lipid-binding assay. Similar results were obtained from three independent experiments. Molecular mass markers (kDa) are indicated on the left.

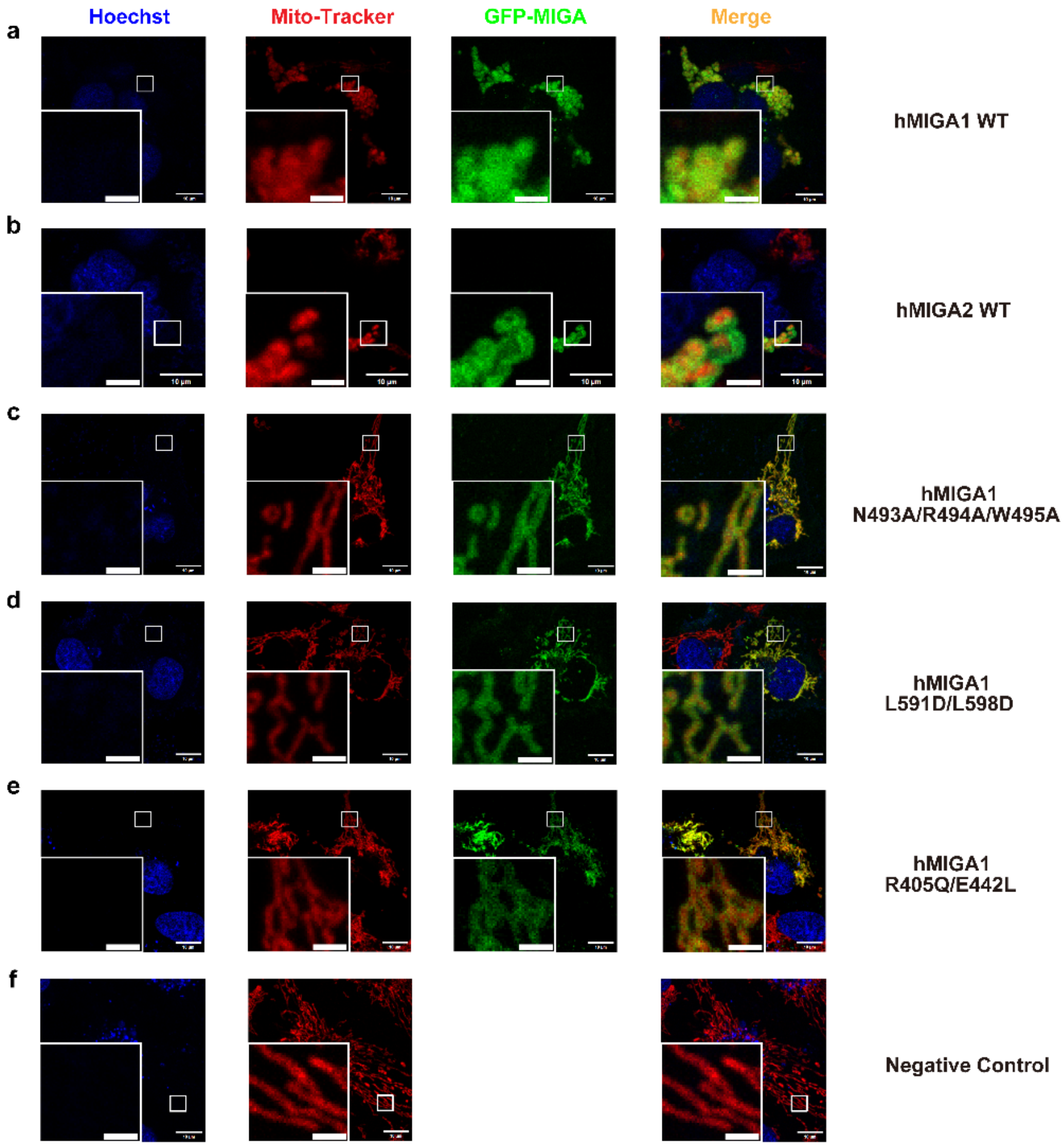

Supplementary Figure S9:

a-e Following the overexpression of GFP-hMIGA1 (**a**) and GFP-hMIGA2 (**b**) wild-type proteins in COS7 cells, fluorescence microscopy revealed that these proteins co-localize with mitochondria exhibiting a highly fused clustered morphology. In contrast, overexpression of various mutant forms of GFP-hMIGA1 (**c-e**) still results in co-localization with mitochondria; however, the proportion of cells exhibiting clustered mitochondria decreases. **f** illustrates the mitochondrial morphology in the control group. Hoechst staining highlights nucleus in blue, while mito-tracker stains the mitochondria in red.

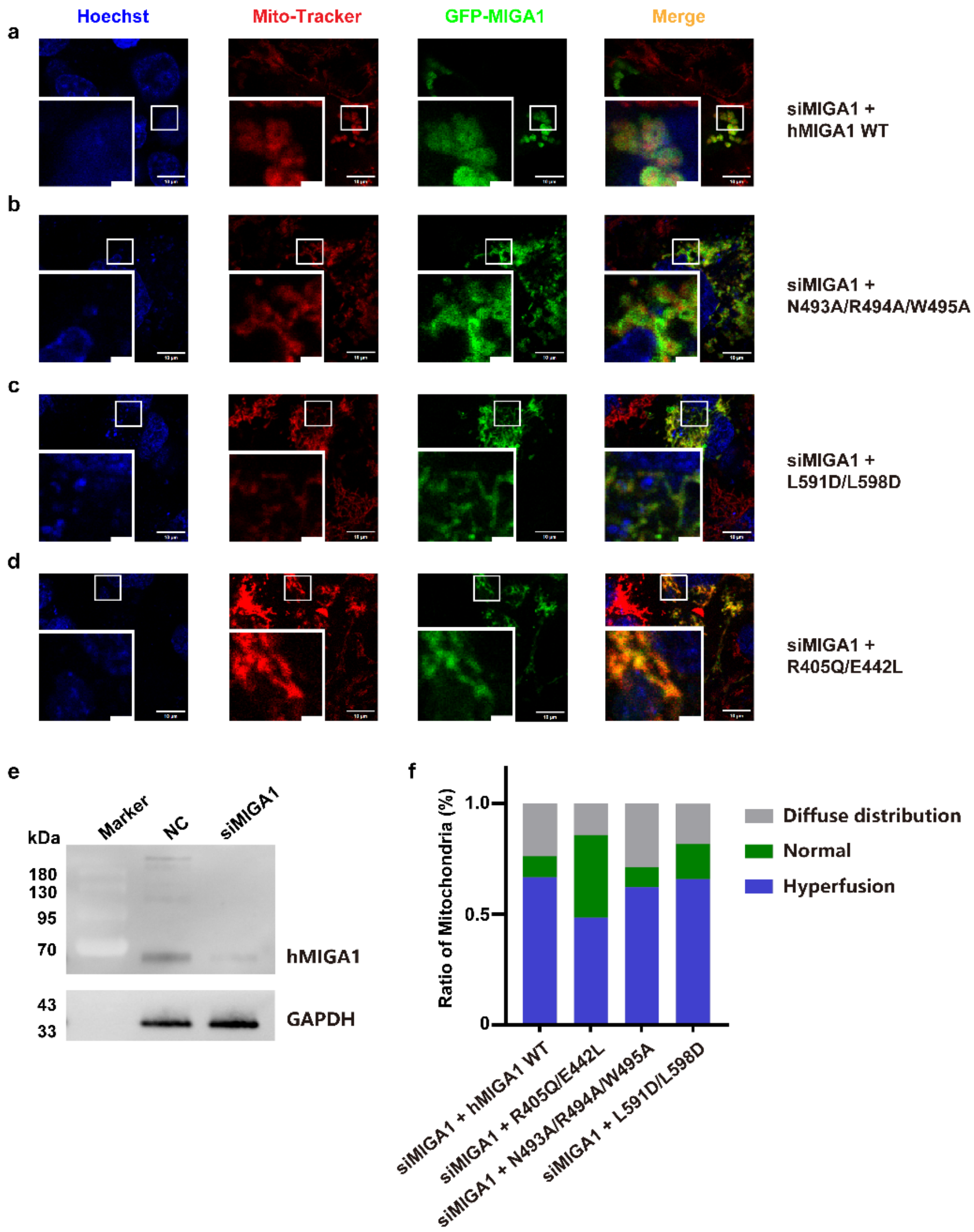

Supplementary Figure S10:

a-d Representative confocal images showing mitochondrial morphology following siRNA-mediated knockdown of endogenous MIGA1 and rescue with GFP-tagged wild-type **(a)** or mutant hMIGA1 constructs (N493A/R494A/W495A mutant for **b**, L591D/L598D mutant for **c** and R405Q/E442L mutant for **d**). Nuclei were stained with Hoechst (blue), mitochondria were labeled with MitoTracker Deep Red (red), and GFP-tagged hMIGA1 is shown in green. Insets show enlarged views of the boxed regions. **e** Western blot analysis confirming knockdown of endogenous hMIGA1 in cells transfected with negative control (NC) or MIGA1 siRNA. GAPDH served as a loading control. **f** Quantification of the percentage of cells exhibiting hyperfused, normal, or diffuse mitochondrial morphology following rescue with wild-type or mutant hMIGA1 constructs. A total of more than 50 cells from three independent biological replicates were analyzed for each group.

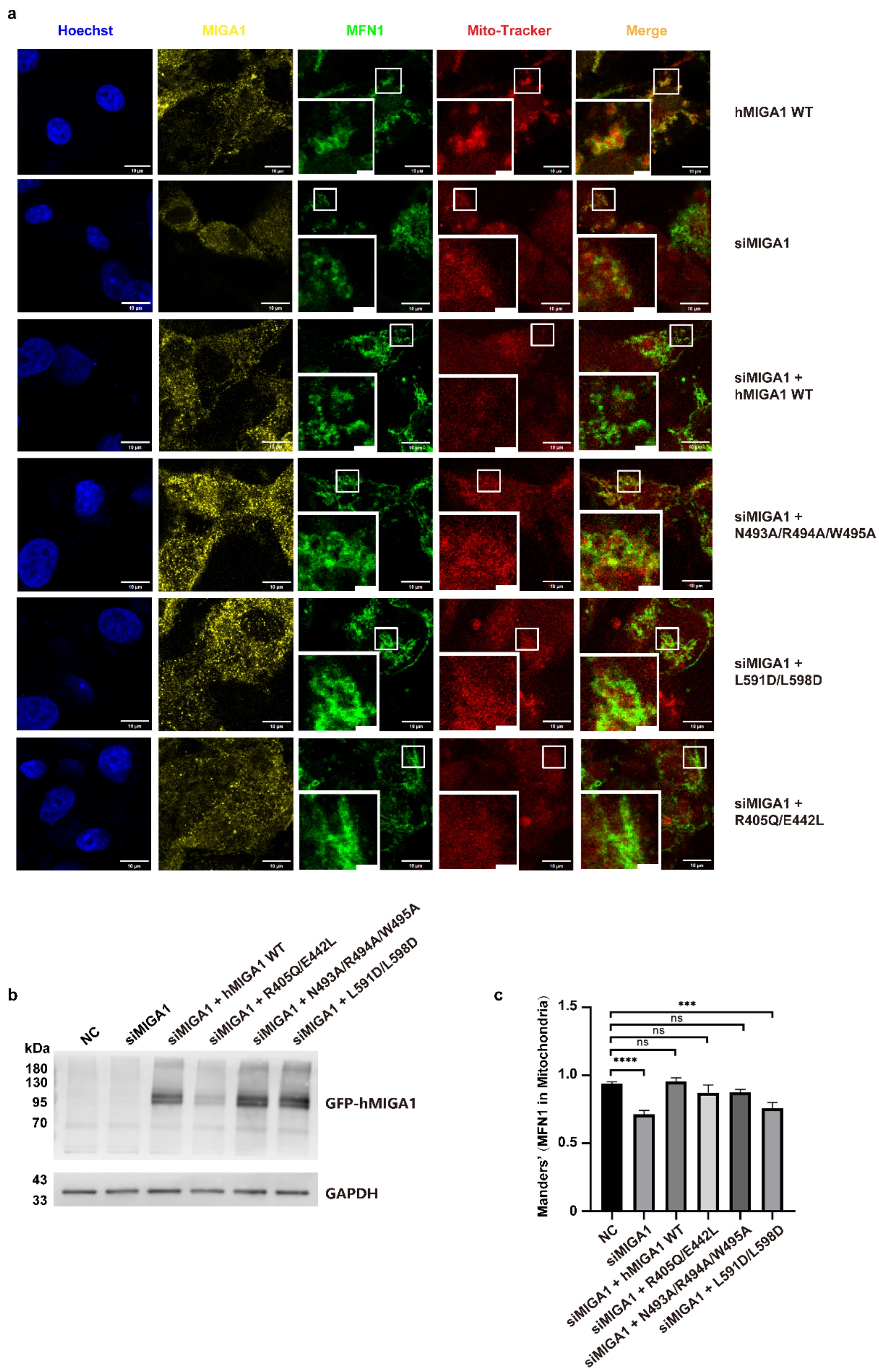

Supplementary Figure S11:

a Representative immunofluorescence images showing the subcellular distribution of hMIGA1 and endogenous MFN1 in the indicated groups. Mitochondria were labeled with MitoTracker Deep Red (red), nuclei were stained with Hoechst (blue), and hMIGA1 and MFN1 are shown in yellow and green, respectively. Insets show enlarged views of the boxed regions. **b** Western blot analysis showing the expression of GFP-tagged wild-type hMIGA1 or the indicated mutants in MIGA1-knockdown cells. GFP-tagged proteins were detected with an anti-GFP antibody. GAPDH served as a loading control. **c** Quantification of the Manders' colocalization coefficient between endogenous MFN1 and mitochondria. Data are presented as mean ± SEM. ***: $p < 0.001$; ****: $p < 0.0001$.

Table S1 Data collection and refinement statistics

hMIGA1 351-606 (PDB ID: 9JCW)	
**Data collection**	
Beamline	BL19U1, SSRF
Temperature (K)	100
Wavelength (Å)	0.97923
Space group	$P2_12_12_1$
Cell parameters	
a, b, c (Å)	88.645, 157.782, 163.907
α, β, γ (°)	90, 90, 90
Resolution (Å)	50.08-2.60 (2.66-2.60)*
Unique reflections	71396 (4518)
R_{merge} (%)	8.0 (122.3)
CC1/2	0.999 (0.757)
I/σI	22.6(2.4)
Completeness (%)	99.9 (99.9)
Redundancy	13.2 (13.4)
**Refinement statistics**	
R_{free} (%)	24.40
R_{work} (%)	21.05
Number of atoms	
Protein	12175
Ligand	235
Water	72
Average B-factors ($Å^2$)	
Protein	81.42
Ligand	87,15
Water	62.46
Root mean square deviations	
Bond lengths (Å)	0.005
Bond angles (°)	0.740
Ramachandran plot, residues in	
Favored regions (%)	97.75
Allowed regions (%)	2.25
Disallowed regions (%)	0

*Highest resolution shell is shown in parenthesis.
