# Supplementary material for: Structural insights into Mitoguardin-1-mediated mitochondrial contact surfaces in fusion and lipid binding
Source: Cell Discov. 2026 Oct 29;12:76. doi: 10.1038/s41421-026-00931-3 (PMC13720014; doi:10.1038/s41421-026-00931-3)
Supplement: Supplementary file 2 [file 41421_2026_931_MOESM3_ESM.pdf]

Full wwPDB X-ray Structure Validation Report (i)

Sep 28, 2024 – 04:09 PM JST

PDB ID	:	9JCW
Title	:	Crystal structure of human MIGA1 LD targeting domain
Deposited on	:	2024-08-30
Resolution	:	2.60 Å(reported)

This wwPDB validation report is for manuscript review

This is a Full wwPDB X-ray Structure Validation Report.

This report is produced by the wwPDB biocuration pipeline after annotation of the structure.

We welcome your comments at *validation@mail.wwpdb.org*
A user guide is available at
https://www.wwpdb.org/validation/2017/XrayValidationReportHelp
with specific help available everywhere you see the (i) symbol.

The types of validation reports are described at
http://www.wwpdb.org/validation/2017/FAQs#types.

For Manuscript Review

The following versions of software and data (see references (i)) were used in the production of this report:

MolProbity	:	4.02b-467
Mogul	:	1.8.5 (274361), CSD as541be (2020)
Xtriage (Phenix)	:	1.13
EDS	:	3.0
buster-report	:	1.1.7 (2018)
Percentile statistics	:	20231227.v01 (using entries in the PDB archive December 27th 2023)
CCP4	:	9.0.003 (Gargrove)
Density-Fitness	:	1.0.11
Ideal geometry (proteins)	:	Engh & Huber (2001)
Ideal geometry (DNA, RNA)	:	Parkinson et al. (1996)

1 Overall quality at a glance (i)

The following experimental techniques were used to determine the structure:
X-RAY DIFFRACTION

The reported resolution of this entry is 2.60 Å.

Percentile scores (ranging between 0-100) for global validation metrics of the entry are shown in the following graphic. The table shows the number of entries on which the scores are based.

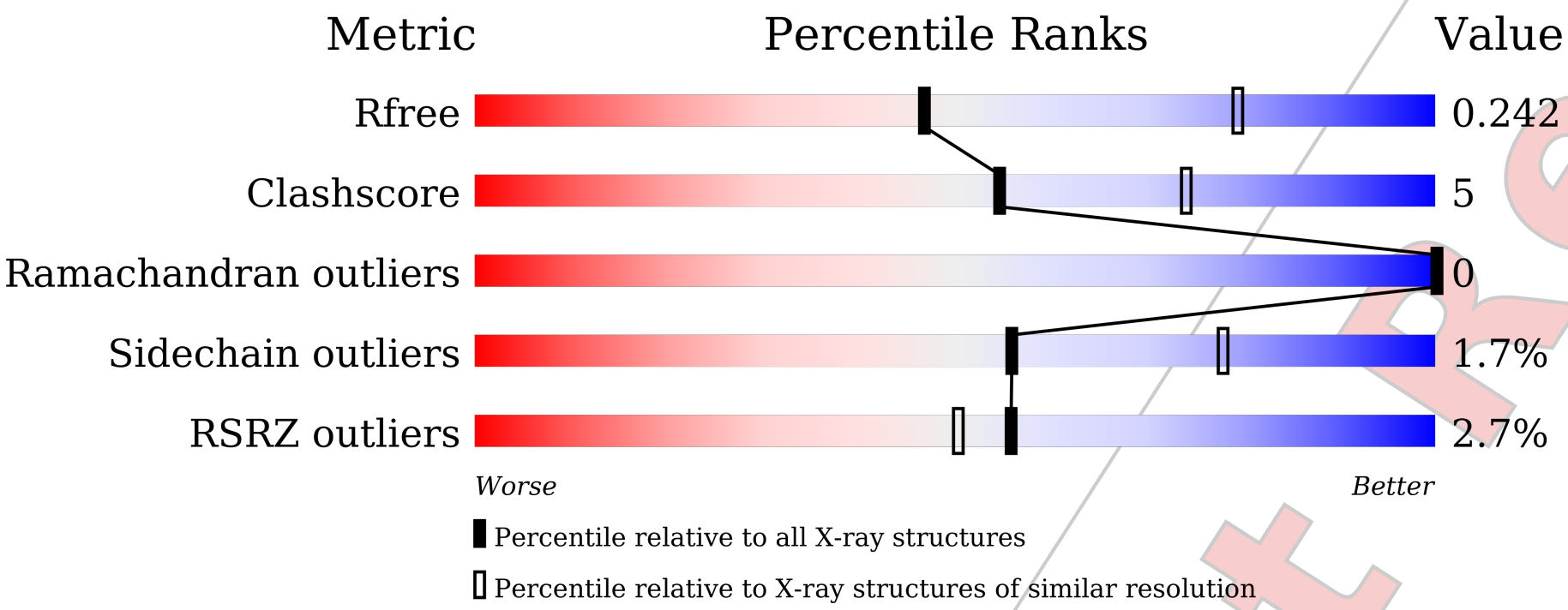

Metric	Whole archive (#Entries)	Similar resolution (#Entries, resolution range(Å))
R_{free}	164625	3775 (2.60-2.60)
Clashscore	180529	4181 (2.60-2.60)
Ramachandran outliers	177936	4129 (2.60-2.60)
Sidechain outliers	177891	4129 (2.60-2.60)
RSRZ outliers	164620	3775 (2.60-2.60)

The table below summarises the geometric issues observed across the polymeric chains and their fit to the electron density. The red, orange, yellow and green segments of the lower bar indicate the fraction of residues that contain outliers for >=3, 2, 1 and 0 types of geometric quality criteria respectively. A grey segment represents the fraction of residues that are not modelled. The numeric value for each fraction is indicated below the corresponding segment, with a dot representing fractions <=5% The upper red bar (where present) indicates the fraction of residues that have poor fit to the electron density. The numeric value is given above the bar.

Mol	Chain	Length	Quality of chain
1	A	258	% / 91% 8% •
1	B	258	87% 12% •
1	C	258	87% 12% •
1	D	258	7% / 82% 13% 5%

Continued on next page...

WORLDWIDE PDB PROTEIN DATA BANK
For Manuscript Review

Continued from previous page...

Mol	Chain	Length	Quality of chain
1	E	258	% 92% 8%
1	F	258	6% 80% 19% •

For Manuscript Review

2 Entry composition (i)

There are 3 unique types of molecules in this entry. The entry contains 12482 atoms, of which 0 are hydrogens and 0 are deuteriums.

In the tables below, the ZeroOcc column contains the number of atoms modelled with zero occupancy, the AltConf column contains the number of residues with at least one atom in alternate conformation and the Trace column contains the number of residues modelled with at most 2 atoms.

- Molecule 1 is a protein called Mitoguardin 1.

Mol	Chain	Residues	Atoms					ZeroOcc	AltConf	Trace
			Total	C	N	O	S			
1	C	256	2079	1342	339	383	15	0	0	0
1	F	255	1993	1283	321	375	14	0	0	0
1	A	256	2076	1339	339	383	15	0	0	0
1	E	258	2085	1346	341	383	15	0	0	0
1	B	256	2079	1342	339	383	15	0	0	0
1	D	245	1863	1192	309	349	13	0	0	0

There are 6 discrepancies between the modelled and reference sequences:

Chain	Residue	Modelled	Actual	Comment	Reference
C	349	MET	-	initiating methionine	UNP Q8NAN2
F	349	MET	-	initiating methionine	UNP Q8NAN2
A	349	MET	-	initiating methionine	UNP Q8NAN2
E	349	MET	-	initiating methionine	UNP Q8NAN2
B	349	MET	-	initiating methionine	UNP Q8NAN2
D	349	MET	-	initiating methionine	UNP Q8NAN2

- Molecule 2 is DI-PALMITOYL-3-SN-PHOSPHATIDYLETHANOLAMINE (three-letter code: PEF) (formula: $C_{37}H_{74}NO_8P$) (labeled as "Ligand of Interest" by depositor).

For Manuscript Review

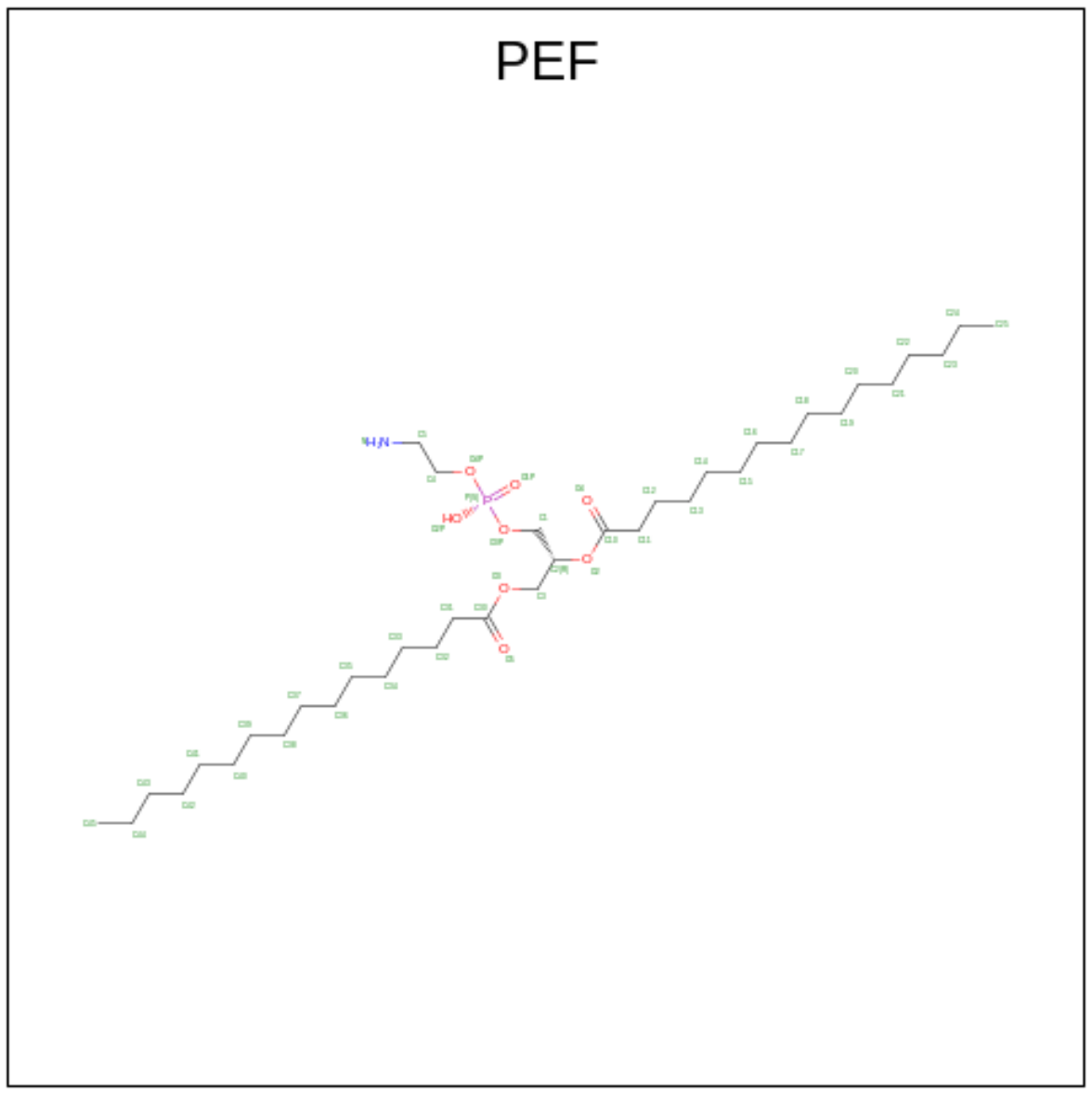

Mol	Chain	Residues	Atoms	ZeroOcc	AltConf
2	C	1	Total C N O P<br>47 37 1 8 1	0	0
2	A	1	Total C N O P<br>47 37 1 8 1	0	0
2	E	1	Total C N O P<br>47 37 1 8 1	0	0
2	B	1	Total C N O P<br>47 37 1 8 1	0	0
2	D	1	Total C N O P<br>47 37 1 8 1	0	0

- Molecule 3 is water.

Mol	Chain	Residues	Atoms	ZeroOcc	AltConf
3	C	12	Total O<br>12 12	0	0
3	F	3	Total O<br>3 3	0	0
3	A	19	Total O<br>19 19	0	0
3	E	22	Total O<br>22 22	0	0
3	B	15	Total O<br>15 15	0	0
3	D	1	Total O<br>1 1	0	0

For Manuscript Review

3 Residue-property plots (i)

These plots are drawn for all protein, RNA, DNA and oligosaccharide chains in the entry. The first graphic for a chain summarises the proportions of the various outlier classes displayed in the second graphic. The second graphic shows the sequence view annotated by issues in geometry and electron density. Residues are color-coded according to the number of geometric quality criteria for which they contain at least one outlier: green = 0, yellow = 1, orange = 2 and red = 3 or more. A red dot above a residue indicates a poor fit to the electron density (RSRZ > 2). Stretches of 2 or more consecutive residues without any outlier are shown as a green connector. Residues present in the sample, but not in the model, are shown in grey.

• Molecule 1: Mitoguardin 1

Chain C: 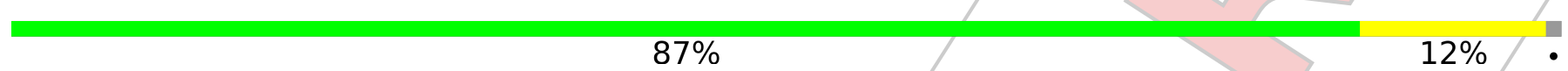

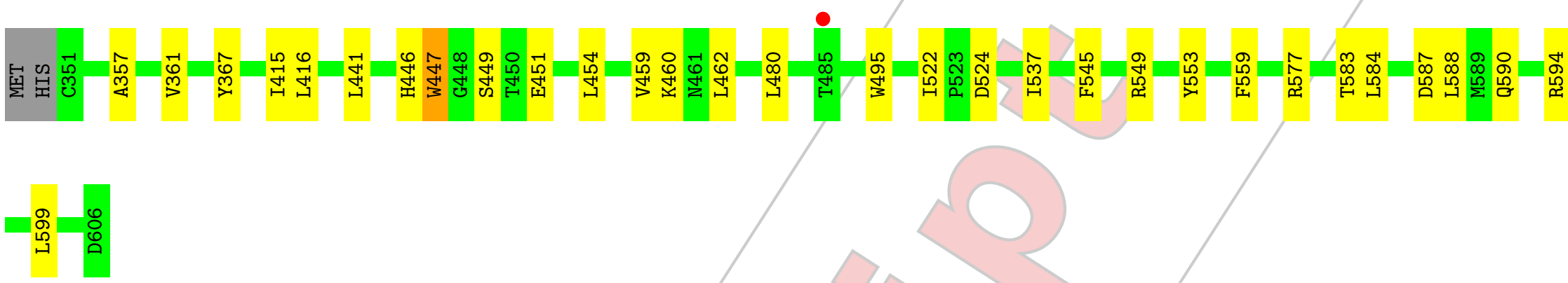

• Molecule 1: Mitoguardin 1

Chain F: 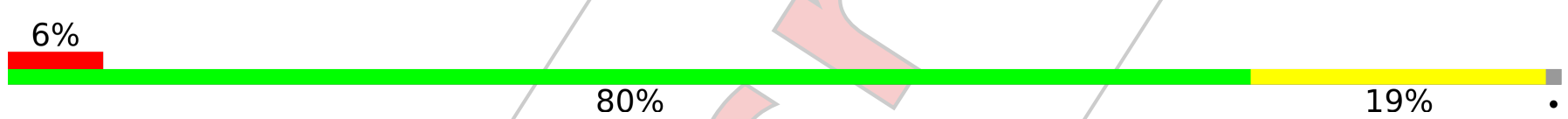

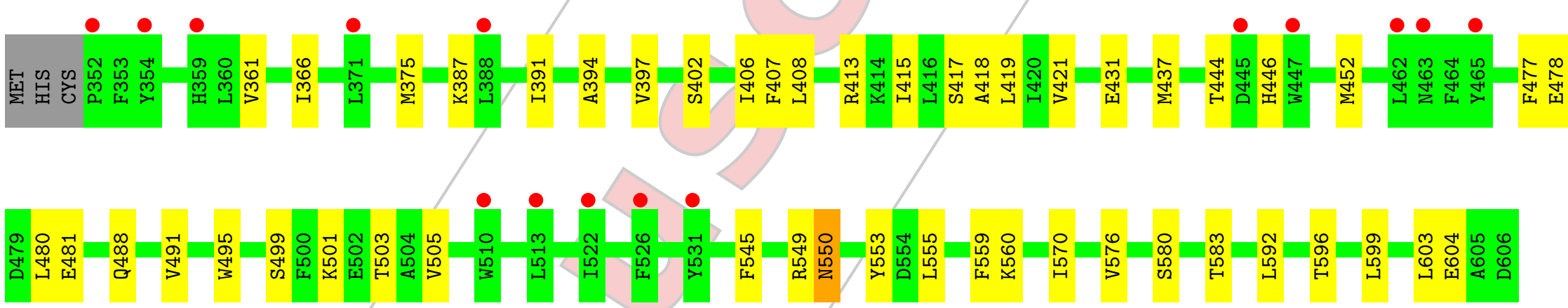

• Molecule 1: Mitoguardin 1

Chain A: 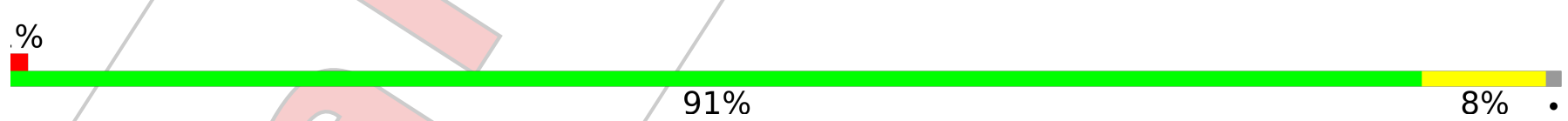

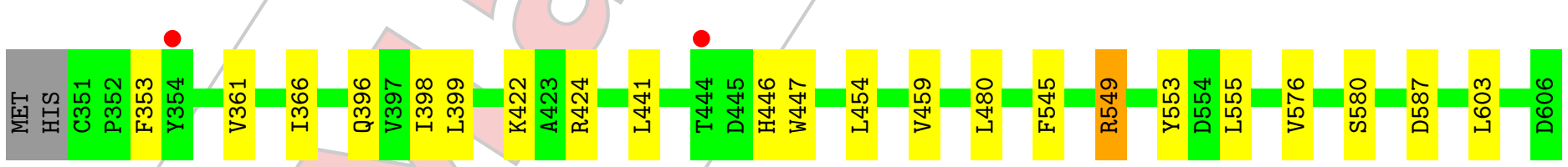

• Molecule 1: Mitoguardin 1

Chain E: 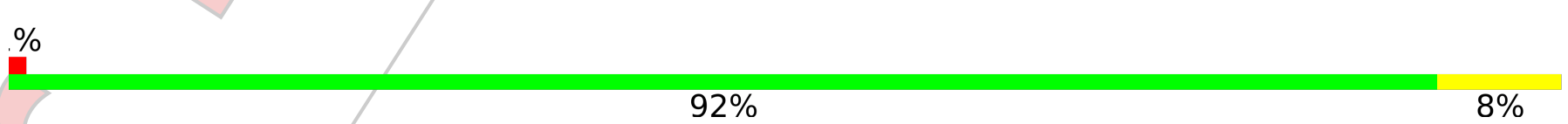

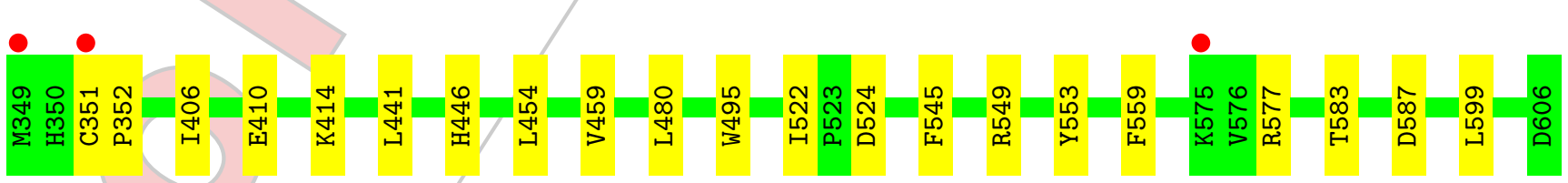

• Molecule 1: Mitoguardin 1

For Manuscript Review

Chain B:

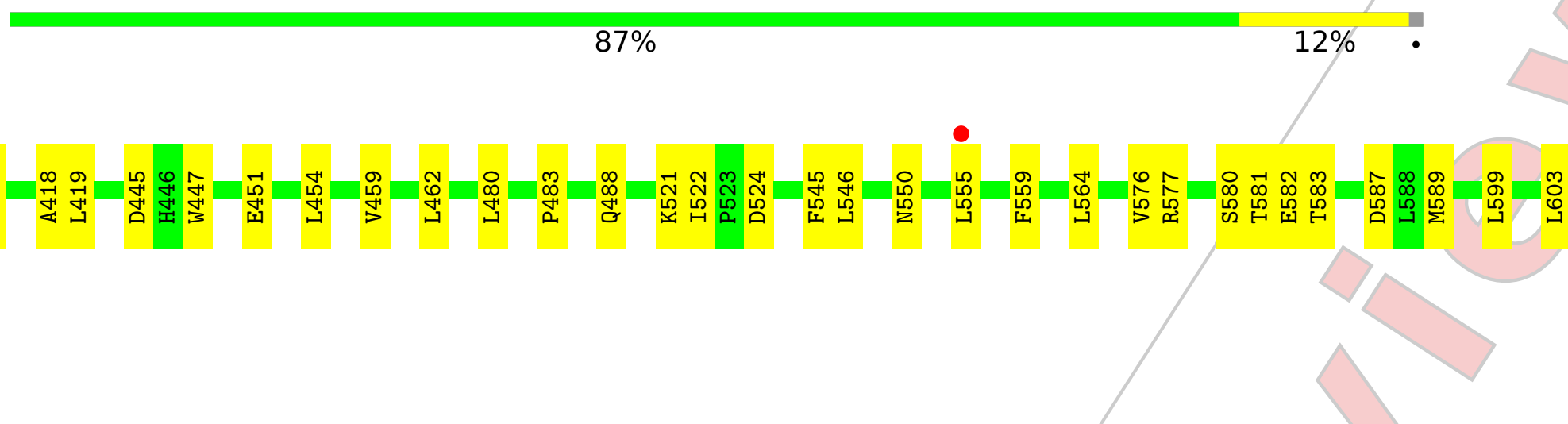

• Molecule 1: Mitoguardin 1

Chain D:

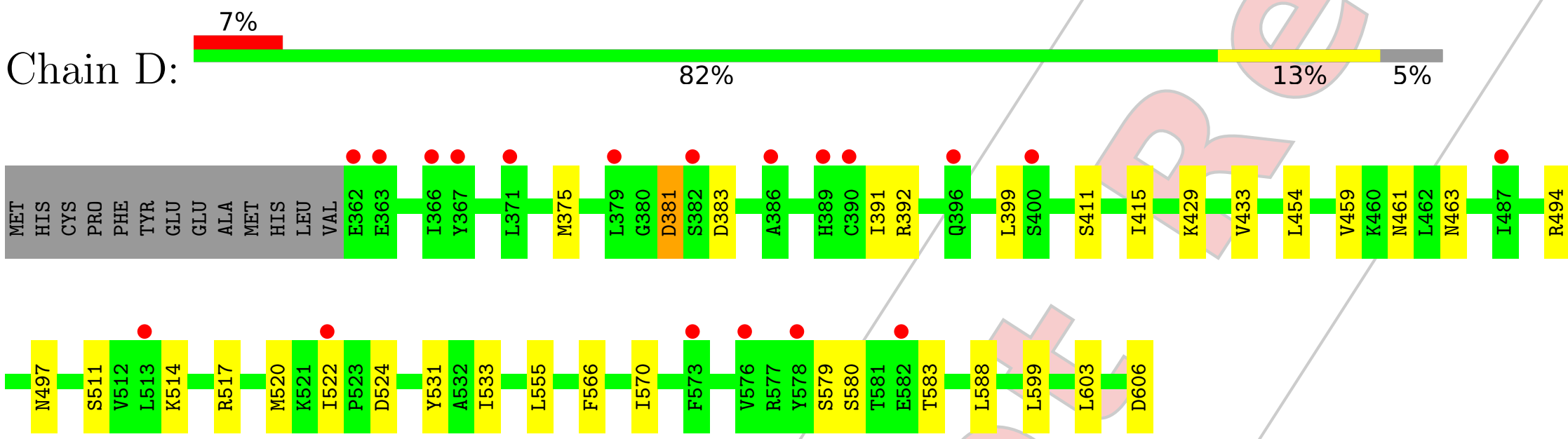

For Manuscript Review

4 Data and refinement statistics (i)

Property	Value	Source
Space group	P 21 21 21	Depositor
Cell constants<br>a, b, c, α, β, γ	88.64Å 157.78Å 163.91Å<br>90.00° 90.00° 90.00°	Depositor
Resolution (Å)	50.08 – 2.60<br>50.08 – 2.60	Depositor<br>EDS
% Data completeness<br>(in resolution range)	99.9 (50.08-2.60)<br>99.9 (50.08-2.60)	Depositor<br>EDS
R_{merge}	(Not available)	Depositor
R_{sym}	(Not available)	Depositor
$< I/\sigma(I) >$ [1]	2.60 (at 2.61Å)	Xtriage
Refinement program	PHENIX (1.19_4092: ???)	Depositor
R, R_{free}	0.210 , 0.244<br>0.209 , 0.242	Depositor<br>DCC
R_{free} test set	3715 reflections (5.20%)	wwPDB-VP
Wilson B-factor (Å^2)	64.1	Xtriage
Anisotropy	0.057	Xtriage
Bulk solvent k_{sol}(e/Å^3), B_{sol}(Å^2)	0.31 , 52.4	EDS
L-test for twinning[2]	$< \|L\| > = 0.49$, $< L^2 > = 0.33$	Xtriage
Estimated twinning fraction	0.005 for -h,l,k	Xtriage
F_o,F_c correlation	0.95	EDS
Total number of atoms	12482	wwPDB-VP
Average B, all atoms (Å^2)	81.0	wwPDB-VP

Xtriage's analysis on translational NCS is as follows: *The largest off-origin peak in the Patterson function is 4.86% of the height of the origin peak. No significant pseudotranslation is detected.*

[1] Intensities estimated from amplitudes.
[2] Theoretical values of $< |L| >$, $< L^2 >$ for acentric reflections are 0.5, 0.333 respectively for untwinned datasets, and 0.375, 0.2 for perfectly twinned datasets.

For Manuscript Review

5 Model quality (i)

5.1 Standard geometry (i)

Bond lengths and bond angles in the following residue types are not validated in this section: PEF

The Z score for a bond length (or angle) is the number of standard deviations the observed value is removed from the expected value. A bond length (or angle) with $|Z| > 5$ is considered an outlier worth inspection. RMSZ is the root-mean-square of all Z scores of the bond lengths (or angles).

Mol	Chain	Bond lengths		Bond angles	
		RMSZ	$\#\|Z\| > 5$	RMSZ	$\#\|Z\| > 5$
1	A	0.27	0/2123	0.45	0/2868
1	B	0.26	0/2126	0.45	0/2872
1	C	0.27	0/2126	0.44	0/2872
1	D	0.31	0/1900	0.49	0/2579
1	E	0.26	0/2132	0.46	0/2881
1	F	0.31	0/2036	0.53	0/2759
All	All	0.28	0/12443	0.47	0/16831

There are no bond length outliers.

There are no bond angle outliers.

There are no chirality outliers.

There are no planarity outliers.

5.2 Too-close contacts (i)

In the following table, the Non-H and H(model) columns list the number of non-hydrogen atoms and hydrogen atoms in the chain respectively. The H(added) column lists the number of hydrogen atoms added and optimized by MolProbity. The Clashes column lists the number of clashes within the asymmetric unit, whereas Symm-Clashes lists symmetry-related clashes.

Mol	Chain	Non-H	H(model)	H(added)	Clashes	Symm-Clashes
1	A	2076	0	2026	12	0
1	B	2079	0	2035	19	0
1	C	2079	0	2035	24	0
1	D	1863	0	1736	21	0
1	E	2085	0	2035	16	0
1	F	1993	0	1887	31	0
2	A	47	0	73	2	0

Continued on next page...

For Manuscript Review

Continued from previous page...

Mol	Chain	Non-H	H(model)	H(added)	Clashes	Symm-Clashes
2	B	47	0	73	0	0
2	C	47	0	73	0	0
2	D	47	0	73	2	0
2	E	47	0	73	0	0
3	A	19	0	0	0	0
3	B	15	0	0	0	0
3	C	12	0	0	0	0
3	D	1	0	0	0	0
3	E	22	0	0	0	0
3	F	3	0	0	0	0
All	All	12482	0	12119	116	0

The all-atom clashscore is defined as the number of clashes found per 1000 atoms (including hydrogen atoms). The all-atom clashscore for this structure is 5.

All (116) close contacts within the same asymmetric unit are listed below, sorted by their clash magnitude.

Atom-1	Atom-2	Interatomic distance (Å)	Clash overlap (Å)
1:B:522:ILE:HG22	1:B:524:ASP:H	1.53	0.73
1:F:413:ARG:HH21	1:F:431:GLU:HG2	1.55	0.72
1:C:451:GLU:CG	1:C:462:LEU:HD12	2.20	0.71
1:C:451:GLU:HG2	1:C:462:LEU:HD12	1.74	0.70
1:F:361:VAL:HG22	1:F:366:ILE:HG13	1.78	0.65
1:F:559:PHE:HA	1:F:599:LEU:HD21	1.79	0.62
1:B:419:LEU:HD23	1:B:555:LEU:HD23	1.83	0.61
1:E:577:ARG:HB2	1:E:583:THR:HG22	1.83	0.60
1:D:411:SER:O	1:D:415:ILE:HG13	2.02	0.60
1:E:522:ILE:HG22	1:E:524:ASP:H	1.67	0.59
1:C:522:ILE:HG22	1:C:524:ASP:H	1.68	0.59
1:E:351:CYS:HB3	1:E:352:PRO:HD3	1.84	0.59
1:C:454:LEU:HB3	1:C:459:VAL:HB	1.85	0.58
1:C:594:ARG:HG3	1:C:594:ARG:HH11	1.69	0.57
1:F:570:ILE:HA	1:F:576:VAL:HG21	1.87	0.57
1:A:555:LEU:HD11	1:A:603:LEU:HD23	1.86	0.57
1:B:581:THR:HG23	1:B:582:GLU:OE2	2.06	0.56
1:D:399:LEU:HD13	2:D:701:PEF:H21	1.86	0.55
1:F:418:ALA:HA	1:F:421:VAL:HG22	1.88	0.55
1:C:451:GLU:HG3	1:C:462:LEU:HD12	1.88	0.54
1:D:517:ARG:HA	1:D:520:MET:HG3	1.89	0.54
1:A:398:ILE:HG22	1:A:399:LEU:HD12	1.89	0.53
1:F:478:GLU:HG3	1:E:495:TRP:CD1	2.44	0.53

Continued on next page...

For Manuscript Review

Continued from previous page...

Atom-1	Atom-2	Interatomic distance (Å)	Clash overlap (Å)
1:F:592:LEU:O	1:F:596:THR:OG1	2.21	0.53
1:B:577:ARG:NE	1:B:583:THR:OG1	2.36	0.53
1:C:549:ARG:HG3	1:C:553:TYR:CE2	2.43	0.53
1:E:454:LEU:HB3	1:E:459:VAL:HB	1.90	0.52
1:C:577:ARG:HG3	1:C:587:ASP:OD2	2.09	0.52
1:E:351:CYS:HB3	1:E:352:PRO:CD	2.38	0.52
1:D:555:LEU:HD21	1:D:603:LEU:HD21	1.91	0.52
1:F:549:ARG:HG3	1:F:553:TYR:CE2	2.44	0.52
1:F:408:LEU:HD11	1:F:592:LEU:HD21	1.92	0.51
1:E:549:ARG:HG3	1:E:553:TYR:CE2	2.45	0.51
1:F:499:SER:O	1:F:503:THR:HG23	2.11	0.51
1:D:555:LEU:HD21	1:D:603:LEU:CD2	2.41	0.50
1:D:392:ARG:HA	1:D:463:ASN:HD21	1.76	0.50
1:F:555:LEU:HD21	1:F:603:LEU:HD12	1.92	0.50
1:B:447:TRP:CZ3	1:B:462:LEU:HD11	2.46	0.50
1:F:417:SER:O	1:F:421:VAL:HG13	2.12	0.49
1:A:447:TRP:CH2	2:A:701:PEF:H21	2.47	0.49
1:D:391:ILE:HG23	1:D:588:LEU:HD11	1.94	0.49
1:C:447:TRP:O	1:C:451:GLU:HG3	2.12	0.49
1:D:579:SER:OG	1:D:583:THR:HG21	2.13	0.49
1:F:488:GLN:HA	1:F:491:VAL:HG22	1.95	0.49
1:F:501:LYS:O	1:F:505:VAL:HG23	2.13	0.48
1:B:454:LEU:HB3	1:B:459:VAL:HB	1.96	0.48
1:A:441:LEU:HA	1:A:446:HIS:NE2	2.28	0.48
1:A:454:LEU:HB3	1:A:459:VAL:HB	1.95	0.48
1:F:480:LEU:HD13	1:F:545:PHE:CD2	2.48	0.48
1:D:566:PHE:O	1:D:570:ILE:HG23	2.14	0.47
1:C:367:TYR:CZ	1:C:460:LYS:HD3	2.49	0.47
1:F:495:TRP:CE3	1:D:375:MET:HE1	2.50	0.47
1:D:517:ARG:HG2	1:D:531:TYR:CD2	2.49	0.47
1:E:406:ILE:O	1:E:410:GLU:HG3	2.14	0.47
1:E:559:PHE:HA	1:E:599:LEU:HD21	1.97	0.47
1:B:559:PHE:HA	1:B:599:LEU:HD21	1.97	0.47
1:F:481:GLU:OE1	1:E:495:TRP:NE1	2.47	0.46
1:B:559:PHE:HD1	1:B:599:LEU:HD11	1.80	0.46
1:D:533:ILE:HG21	2:D:701:PEF:H392	1.98	0.46
1:D:579:SER:OG	1:D:580:SER:N	2.46	0.46
1:F:413:ARG:HE	1:F:431:GLU:CG	2.28	0.46
1:F:419:LEU:HD22	1:F:555:LEU:HD11	1.98	0.46
1:C:416:LEU:HB3	1:C:537:ILE:HD13	1.97	0.46
1:B:447:TRP:O	1:B:451:GLU:HG3	2.16	0.46

Continued on next page...

For Manuscript Review

Continued from previous page...

Atom-1	Atom-2	Interatomic distance (Å)	Clash overlap (Å)
1:E:559:PHE:HD1	1:E:599:LEU:HD11	1.80	0.46
1:B:580:SER:H	1:B:583:THR:HG22	1.80	0.45
1:A:576:VAL:HG22	1:A:587:ASP:HB3	1.99	0.45
1:F:477:PHE:CD1	1:F:560:LYS:HG3	2.52	0.45
1:D:599:LEU:O	1:D:603:LEU:HG	2.17	0.45
1:F:387:LYS:O	1:F:391:ILE:HG13	2.17	0.45
1:B:483:PRO:HG3	1:B:546:LEU:HD21	1.99	0.44
1:D:429:LYS:O	1:D:433:VAL:HG22	2.17	0.44
1:D:381:ASP:C	1:D:383:ASP:H	2.21	0.44
1:E:549:ARG:HG3	1:E:553:TYR:CD2	2.53	0.44
1:E:577:ARG:HG3	1:E:587:ASP:OD2	2.17	0.44
1:D:392:ARG:HA	1:D:463:ASN:ND2	2.33	0.44
1:F:550:ASN:H	1:F:550:ASN:HD22	1.66	0.44
1:A:353:PHE:HB2	1:A:580:SER:HA	1.99	0.43
1:C:480:LEU:HD13	1:C:545:PHE:CD2	2.53	0.43
1:E:441:LEU:HA	1:E:446:HIS:NE2	2.33	0.43
1:C:584:LEU:O	1:C:588:LEU:HD23	2.18	0.43
1:B:418:ALA:CB	1:B:603:LEU:HD22	2.48	0.43
1:C:590:GLN:O	1:C:594:ARG:HG2	2.19	0.43
1:C:441:LEU:HA	1:C:446:HIS:NE2	2.34	0.42
1:F:580:SER:H	1:F:583:THR:CG2	2.32	0.42
1:C:594:ARG:HD2	1:F:604:GLU:HB3	2.02	0.42
1:B:576:VAL:HG12	1:B:587:ASP:HB3	2.02	0.42
1:F:375:MET:HG3	1:E:495:TRP:CE3	2.55	0.42
1:B:521:LYS:C	1:B:522:ILE:HD13	2.39	0.42
1:F:413:ARG:HE	1:F:431:GLU:HG3	1.84	0.42
1:D:399:LEU:HD23	1:D:399:LEU:HA	1.85	0.42
1:A:361:VAL:HG22	1:A:366:ILE:HG13	2.02	0.41
1:E:480:LEU:HD13	1:E:545:PHE:CD2	2.55	0.41
1:B:577:ARG:HG3	1:B:587:ASP:OD2	2.21	0.41
1:F:415:ILE:O	1:F:419:LEU:HD23	2.20	0.41
1:A:447:TRP:HH2	2:A:701:PEF:H21	1.85	0.41
1:C:357:ALA:O	1:C:361:VAL:HG23	2.19	0.41
1:F:406:ILE:HD12	1:F:407:PHE:N	2.35	0.41
1:B:353:PHE:HB2	1:B:580:SER:HA	2.01	0.41
1:B:555:LEU:HD21	1:B:603:LEU:HD23	2.01	0.41
1:A:480:LEU:HD13	1:A:545:PHE:CD2	2.55	0.41
1:B:480:LEU:HD13	1:B:545:PHE:CD2	2.55	0.41
1:D:454:LEU:HB3	1:D:459:VAL:HB	2.02	0.41
1:D:522:ILE:HG22	1:D:524:ASP:H	1.85	0.41
1:C:577:ARG:NE	1:C:583:THR:OG1	2.43	0.41

Continued on next page...

For Manuscript Review

Continued from previous page...

Atom-1	Atom-2	Interatomic distance (Å)	Clash overlap (Å)
1:A:422:LYS:HA	1:A:422:LYS:HD3	1.94	0.41
1:A:549:ARG:HG3	1:A:553:TYR:CE2	2.55	0.41
1:C:415:ILE:HD13	1:C:599:LEU:HD13	2.03	0.41
1:C:559:PHE:HD1	1:C:599:LEU:HD11	1.86	0.41
1:C:416:LEU:HB3	1:C:537:ILE:CD1	2.52	0.40
1:C:549:ARG:HG3	1:C:553:TYR:CD2	2.56	0.40
1:C:367:TYR:CE2	1:C:460:LYS:HD3	2.56	0.40
1:F:495:TRP:HE3	1:D:375:MET:HE1	1.86	0.40
1:C:495:TRP:HH2	1:B:564:LEU:HD21	1.86	0.40
1:F:394:ALA:O	1:F:397:VAL:HG22	2.21	0.40
1:F:444:THR:O	1:F:446:HIS:HD2	2.04	0.40

There are no symmetry-related clashes.

5.3 Torsion angles (i)

5.3.1 Protein backbone (i)

In the following table, the Percentiles column shows the percent Ramachandran outliers of the chain as a percentile score with respect to all X-ray entries followed by that with respect to entries of similar resolution.

The Analysed column shows the number of residues for which the backbone conformation was analysed, and the total number of residues.

Mol	Chain	Analysed	Favoured	Allowed	Outliers	Percentiles	
1	A	254/258 (98%)	250 (98%)	4 (2%)	0	100	100
1	B	254/258 (98%)	250 (98%)	4 (2%)	0	100	100
1	C	254/258 (98%)	249 (98%)	5 (2%)	0	100	100
1	D	243/258 (94%)	237 (98%)	6 (2%)	0	100	100
1	E	256/258 (99%)	249 (97%)	7 (3%)	0	100	100
1	F	253/258 (98%)	245 (97%)	8 (3%)	0	100	100
All	All	1514/1548 (98%)	1480 (98%)	34 (2%)	0	100	100

There are no Ramachandran outliers to report.

For Manuscript Review

5.3.2 Protein sidechains (i)

In the following table, the Percentiles column shows the percent sidechain outliers of the chain as a percentile score with respect to all X-ray entries followed by that with respect to entries of similar resolution.

The Analysed column shows the number of residues for which the sidechain conformation was analysed, and the total number of residues.

Mol	Chain	Analysed	Rotameric	Outliers	Percentiles	
1	A	226/233 (97%)	223 (99%)	3 (1%)	65	84
1	B	227/233 (97%)	222 (98%)	5 (2%)	47	72
1	C	227/233 (97%)	225 (99%)	2 (1%)	75	90
1	D	189/233 (81%)	182 (96%)	7 (4%)	29	55
1	E	226/233 (97%)	225 (100%)	1 (0%)	89	96
1	F	209/233 (90%)	205 (98%)	4 (2%)	52	75
All	All	1304/1398 (93%)	1282 (98%)	22 (2%)	56	78

All (22) residues with a non-rotameric sidechain are listed below:

Mol	Chain	Res	Type
1	C	447	TRP
1	C	449	SER
1	F	402	SER
1	F	437	MET
1	F	452	MET
1	F	550	ASN
1	A	396	GLN
1	A	424	ARG
1	A	549	ARG
1	E	414	LYS
1	B	368	SER
1	B	445	ASP
1	B	488	GLN
1	B	550	ASN
1	B	589	MET
1	D	381	ASP
1	D	461	ASN
1	D	494	ARG
1	D	497	ASN
1	D	511	SER
1	D	514	LYS
1	D	606	ASP

For Manuscript Review

Sometimes sidechains can be flipped to improve hydrogen bonding and reduce clashes. There are no such sidechains identified.

5.3.3 RNA (i)

There are no RNA molecules in this entry.

5.4 Non-standard residues in protein, DNA, RNA chains (i)

There are no non-standard protein/DNA/RNA residues in this entry.

5.5 Carbohydrates (i)

There are no oligosaccharides in this entry.

5.6 Ligand geometry (i)

5 ligands are modelled in this entry.

In the following table, the Counts columns list the number of bonds (or angles) for which Mogul statistics could be retrieved, the number of bonds (or angles) that are observed in the model and the number of bonds (or angles) that are defined in the Chemical Component Dictionary. The Link column lists molecule types, if any, to which the group is linked. The Z score for a bond length (or angle) is the number of standard deviations the observed value is removed from the expected value. A bond length (or angle) with $|Z| > 2$ is considered an outlier worth inspection. RMSZ is the root-mean-square of all Z scores of the bond lengths (or angles).

Mol	Type	Chain	Res	Link	Bond lengths			Bond angles		
					Counts	RMSZ	$\#\|Z\| > 2$	Counts	RMSZ	$\#\|Z\| > 2$
2	PEF	D	701	-	46,46,46	1.01	3 (6%)	49,51,51	1.02	4 (8%)
2	PEF	A	701	-	46,46,46	0.29	0	49,51,51	0.36	0
2	PEF	B	701	-	46,46,46	0.29	0	49,51,51	0.38	0
2	PEF	C	701	-	46,46,46	0.29	0	49,51,51	0.31	0
2	PEF	E	701	-	46,46,46	1.05	3 (6%)	49,51,51	0.93	2 (4%)

In the following table, the Chirals column lists the number of chiral outliers, the number of chiral centers analysed, the number of these observed in the model and the number defined in the Chemical Component Dictionary. Similar counts are reported in the Torsion and Rings columns. '-' means no outliers of that kind were identified.

Mol	Type	Chain	Res	Link	Chirals	Torsions	Rings
2	PEF	D	701	-	-	25/50/50/50	-

Continued on next page...

For Manuscript Review

Continued from previous page...

Mol	Type	Chain	Res	Link	Chirals	Torsions	Rings
2	PEF	A	701	-	-	28/50/50/50	-
2	PEF	B	701	-	-	26/50/50/50	-
2	PEF	C	701	-	-	26/50/50/50	-
2	PEF	E	701	-	-	18/50/50/50	-

All (6) bond length outliers are listed below:

Mol	Chain	Res	Type	Atoms	Z	Observed(Å)	Ideal(Å)
2	E	701	PEF	O3-C30	3.01	1.42	1.33
2	D	701	PEF	O3-C30	2.96	1.42	1.33
2	E	701	PEF	O2-C10	2.91	1.42	1.34
2	D	701	PEF	O2-C2	-2.89	1.39	1.46
2	D	701	PEF	O2-C10	2.51	1.41	1.34
2	E	701	PEF	O2-C2	-2.44	1.40	1.46

All (6) bond angle outliers are listed below:

Mol	Chain	Res	Type	Atoms	Z	Observed(^{o})	Ideal(^{o})
2	D	701	PEF	O2-C10-C11	4.34	120.84	111.50
2	E	701	PEF	O2-C10-C11	4.31	120.80	111.50
2	D	701	PEF	C2-O2-C10	-3.12	110.10	117.79
2	E	701	PEF	O3-C30-C31	2.62	120.12	111.91
2	D	701	PEF	O3-C30-C31	2.35	119.29	111.91
2	D	701	PEF	O2-C10-O4	-2.03	118.79	123.70

There are no chirality outliers.

All (123) torsion outliers are listed below:

Mol	Chain	Res	Type	Atoms
2	C	701	PEF	O4P-C4-C5-N
2	C	701	PEF	C1-O3P-P-O1P
2	C	701	PEF	C4-O4P-P-O1P
2	A	701	PEF	O4P-C4-C5-N
2	A	701	PEF	C11-C10-O2-C2
2	A	701	PEF	C1-O3P-P-O1P
2	A	701	PEF	C1-O3P-P-O2P
2	E	701	PEF	O4P-C4-C5-N
2	E	701	PEF	C1-O3P-P-O1P
2	B	701	PEF	O4P-C4-C5-N
2	B	701	PEF	C11-C10-O2-C2

Continued on next page...

For Manuscript Review

Continued from previous page...

Mol	Chain	Res	Type	Atoms
2	B	701	PEF	C31-C30-O3-C3
2	B	701	PEF	O5-C30-O3-C3
2	B	701	PEF	C1-O3P-P-O2P
2	D	701	PEF	O4P-C4-C5-N
2	D	701	PEF	C1-O3P-P-O1P
2	D	701	PEF	C1-O3P-P-O2P
2	D	701	PEF	C1-O3P-P-O4P
2	B	701	PEF	O4-C10-O2-C2
2	D	701	PEF	C14-C15-C16-C17
2	D	701	PEF	C41-C42-C43-C44
2	E	701	PEF	C31-C30-O3-C3
2	A	701	PEF	O4-C10-O2-C2
2	E	701	PEF	O5-C30-O3-C3
2	B	701	PEF	C21-C22-C23-C24
2	C	701	PEF	C10-C11-C12-C13
2	C	701	PEF	C30-C31-C32-C33
2	E	701	PEF	C30-C31-C32-C33
2	C	701	PEF	C1-O3P-P-O4P
2	A	701	PEF	C1-O3P-P-O4P
2	A	701	PEF	C4-O4P-P-O3P
2	B	701	PEF	C1-O3P-P-O4P
2	B	701	PEF	C35-C36-C37-C38
2	C	701	PEF	C11-C10-O2-C2
2	A	701	PEF	C34-C35-C36-C37
2	A	701	PEF	C37-C38-C39-C40
2	B	701	PEF	C36-C37-C38-C39
2	C	701	PEF	C18-C19-C20-C21
2	A	701	PEF	C39-C40-C41-C42
2	A	701	PEF	C41-C42-C43-C44
2	E	701	PEF	C19-C20-C21-C22
2	D	701	PEF	C19-C20-C21-C22
2	D	701	PEF	C20-C21-C22-C23
2	C	701	PEF	O4-C10-O2-C2
2	C	701	PEF	C17-C18-C19-C20
2	A	701	PEF	C16-C17-C18-C19
2	D	701	PEF	C21-C22-C23-C24
2	A	701	PEF	C33-C34-C35-C36
2	A	701	PEF	C38-C39-C40-C41
2	E	701	PEF	C34-C35-C36-C37
2	B	701	PEF	C15-C16-C17-C18
2	D	701	PEF	C12-C13-C14-C15
2	E	701	PEF	C41-C42-C43-C44

Continued on next page...

For Manuscript Review

Continued from previous page...

Mol	Chain	Res	Type	Atoms
2	D	701	PEF	C35-C36-C37-C38
2	A	701	PEF	C21-C22-C23-C24
2	D	701	PEF	C11-C12-C13-C14
2	A	701	PEF	C32-C33-C34-C35
2	E	701	PEF	C36-C37-C38-C39
2	A	701	PEF	C36-C37-C38-C39
2	C	701	PEF	C22-C23-C24-C25
2	E	701	PEF	C17-C18-C19-C20
2	C	701	PEF	C35-C36-C37-C38
2	C	701	PEF	C40-C41-C42-C43
2	C	701	PEF	C20-C21-C22-C23
2	C	701	PEF	C13-C14-C15-C16
2	D	701	PEF	C30-C31-C32-C33
2	A	701	PEF	C31-C30-O3-C3
2	B	701	PEF	C20-C21-C22-C23
2	A	701	PEF	C10-C11-C12-C13
2	C	701	PEF	C33-C34-C35-C36
2	D	701	PEF	C11-C10-O2-C2
2	D	701	PEF	O4-C10-O2-C2
2	A	701	PEF	O2-C2-C3-O3
2	E	701	PEF	C10-C11-C12-C13
2	C	701	PEF	O3P-C1-C2-C3
2	B	701	PEF	O3P-C1-C2-C3
2	B	701	PEF	C33-C34-C35-C36
2	A	701	PEF	C35-C36-C37-C38
2	A	701	PEF	C17-C18-C19-C20
2	A	701	PEF	C1-C2-C3-O3
2	A	701	PEF	O5-C30-O3-C3
2	B	701	PEF	C11-C12-C13-C14
2	D	701	PEF	C22-C23-C24-C25
2	B	701	PEF	C34-C35-C36-C37
2	B	701	PEF	C41-C42-C43-C44
2	B	701	PEF	O2-C2-C3-O3
2	C	701	PEF	C41-C42-C43-C44
2	B	701	PEF	C16-C17-C18-C19
2	D	701	PEF	C37-C38-C39-C40
2	C	701	PEF	C2-C1-O3P-P
2	A	701	PEF	C12-C13-C14-C15
2	E	701	PEF	C16-C17-C18-C19
2	E	701	PEF	C42-C43-C44-C45
2	D	701	PEF	C40-C41-C42-C43
2	B	701	PEF	C10-C11-C12-C13

Continued on next page...

For Manuscript Review

Continued from previous page...

Mol	Chain	Res	Type	Atoms
2	C	701	PEF	O3P-C1-C2-O2
2	B	701	PEF	O3P-C1-C2-O2
2	E	701	PEF	O2-C2-C3-O3
2	D	701	PEF	C31-C32-C33-C34
2	D	701	PEF	C18-C19-C20-C21
2	C	701	PEF	C14-C15-C16-C17
2	D	701	PEF	C10-C11-C12-C13
2	A	701	PEF	C22-C23-C24-C25
2	C	701	PEF	C1-O3P-P-O2P
2	A	701	PEF	C4-O4P-P-O1P
2	B	701	PEF	C1-O3P-P-O1P
2	A	701	PEF	C11-C12-C13-C14
2	B	701	PEF	C1-C2-C3-O3
2	C	701	PEF	C15-C16-C17-C18
2	E	701	PEF	C18-C19-C20-C21
2	C	701	PEF	C4-O4P-P-O3P
2	B	701	PEF	C4-O4P-P-O3P
2	E	701	PEF	C1-C2-C3-O3
2	C	701	PEF	C32-C33-C34-C35
2	B	701	PEF	C22-C23-C24-C25
2	D	701	PEF	O3-C30-C31-C32
2	D	701	PEF	C13-C14-C15-C16
2	B	701	PEF	C40-C41-C42-C43
2	C	701	PEF	C36-C37-C38-C39
2	D	701	PEF	C17-C18-C19-C20
2	D	701	PEF	C42-C43-C44-C45
2	E	701	PEF	C11-C12-C13-C14
2	E	701	PEF	O3-C30-C31-C32

There are no ring outliers.

2 monomers are involved in 4 short contacts:

Mol	Chain	Res	Type	Clashes	Symm-Clashes
2	D	701	PEF	2	0
2	A	701	PEF	2	0

The following is a two-dimensional graphical depiction of Mogul quality analysis of bond lengths, bond angles, torsion angles, and ring geometry for all instances of the Ligand of Interest. In addition, ligands with molecular weight > 250 and outliers as shown on the validation Tables will also be included. For torsion angles, if less then 5% of the Mogul distribution of torsion angles is within 10 degrees of the torsion angle in question, then that torsion angle is considered an outlier. Any bond that is central to one or more torsion angles identified as an outlier by Mogul will be

For Manuscript Review

highlighted in the graph. For rings, the root-mean-square deviation (RMSD) between the ring in question and similar rings identified by Mogul is calculated over all ring torsion angles. If the average RMSD is greater than 60 degrees and the minimal RMSD between the ring in question and any Mogul-identified rings is also greater than 60 degrees, then that ring is considered an outlier. The outliers are highlighted in purple. The color gray indicates Mogul did not find sufficient equivalents in the CSD to analyse the geometry.

Ligand PEF D 701

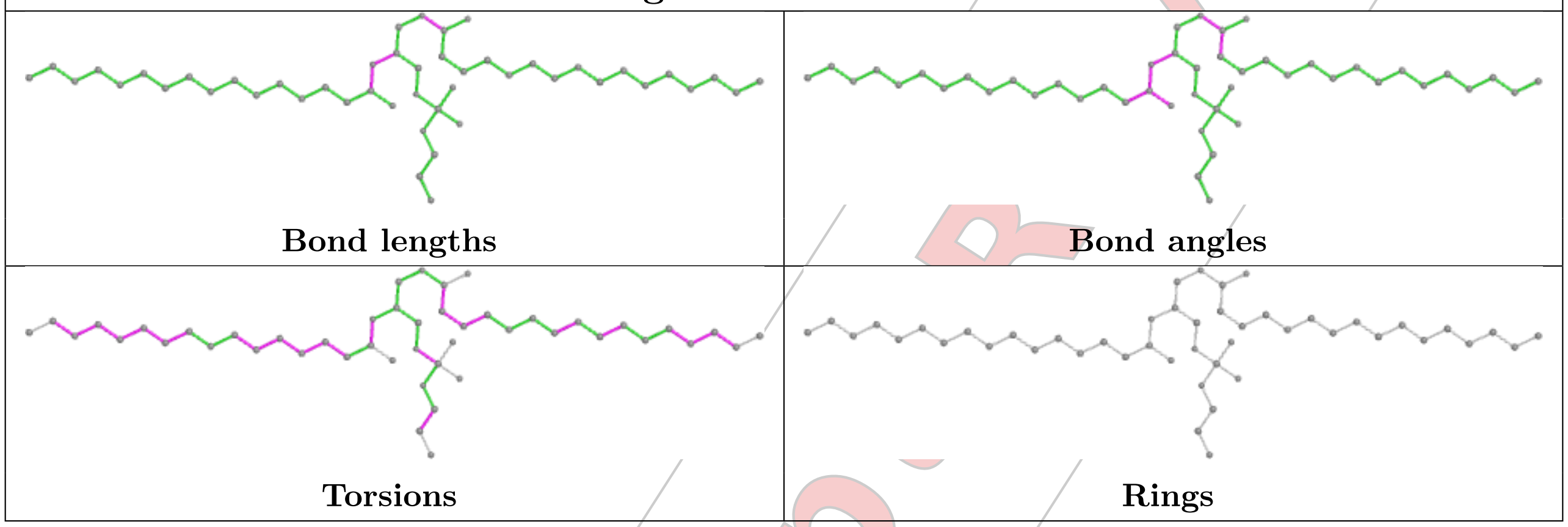

Ligand PEF A 701

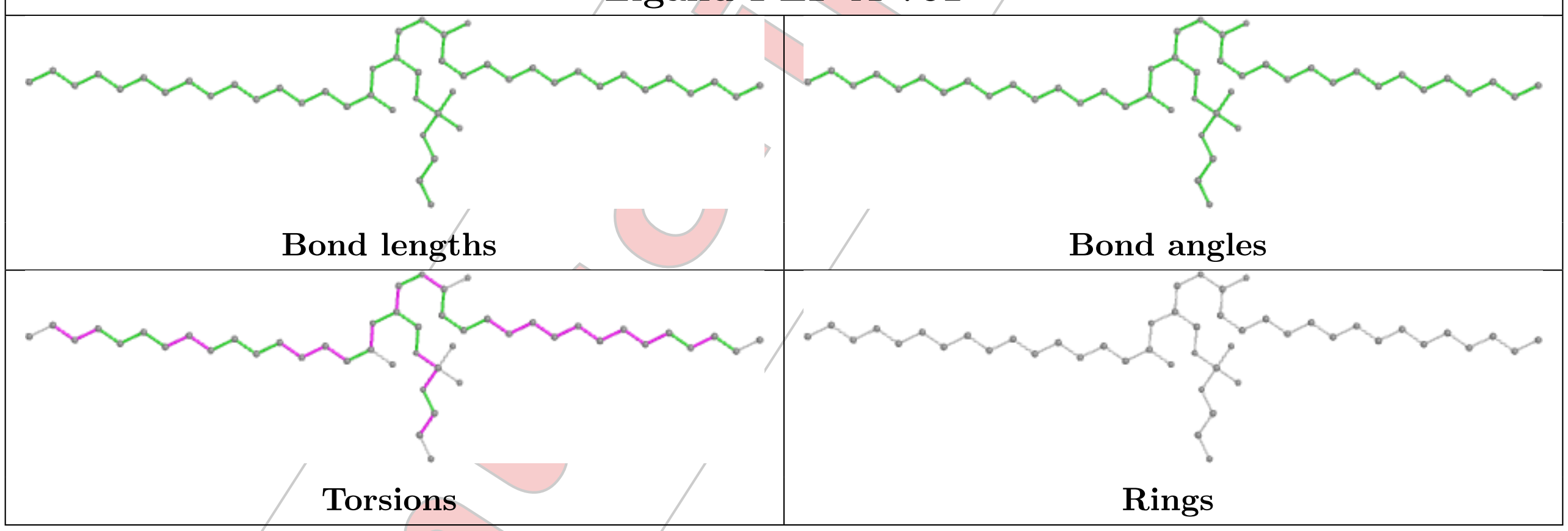

Ligand PEF B 701

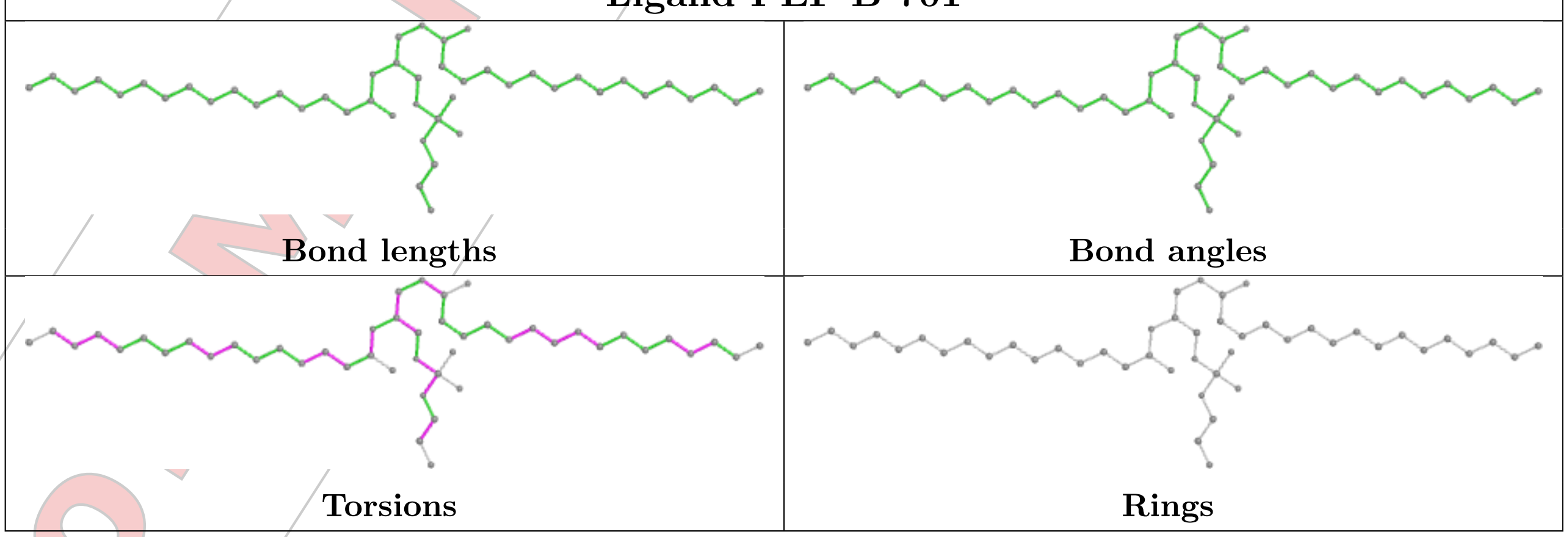

For Manuscript Review

Ligand PEF C 701

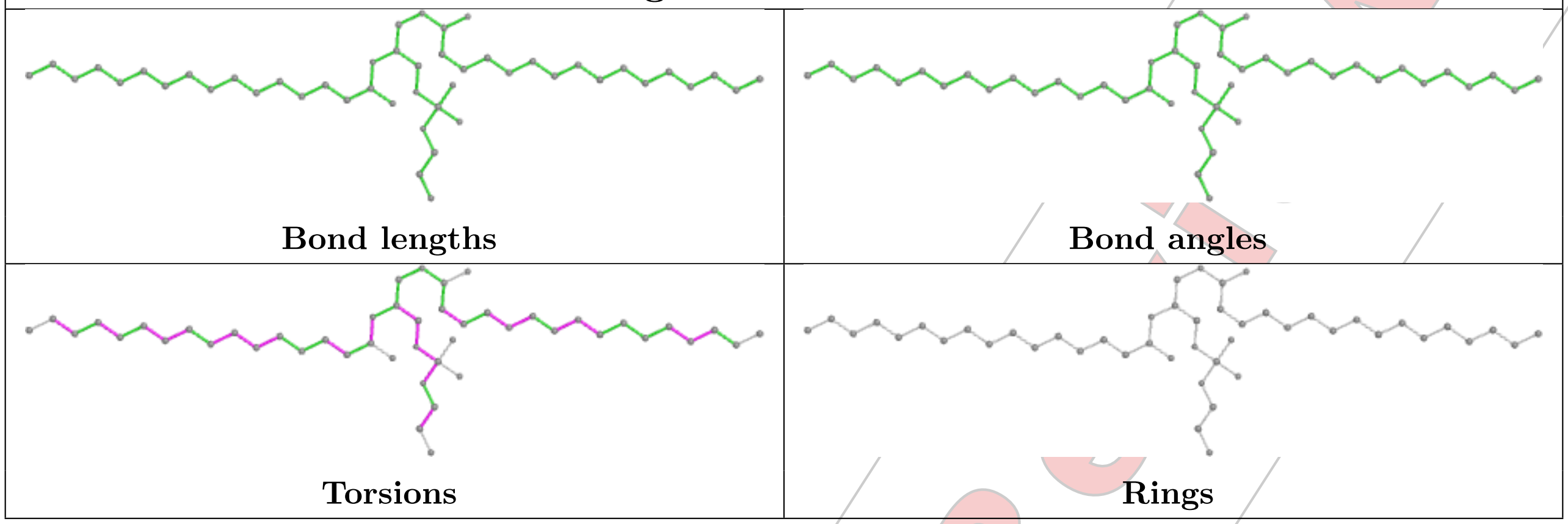

Ligand PEF E 701

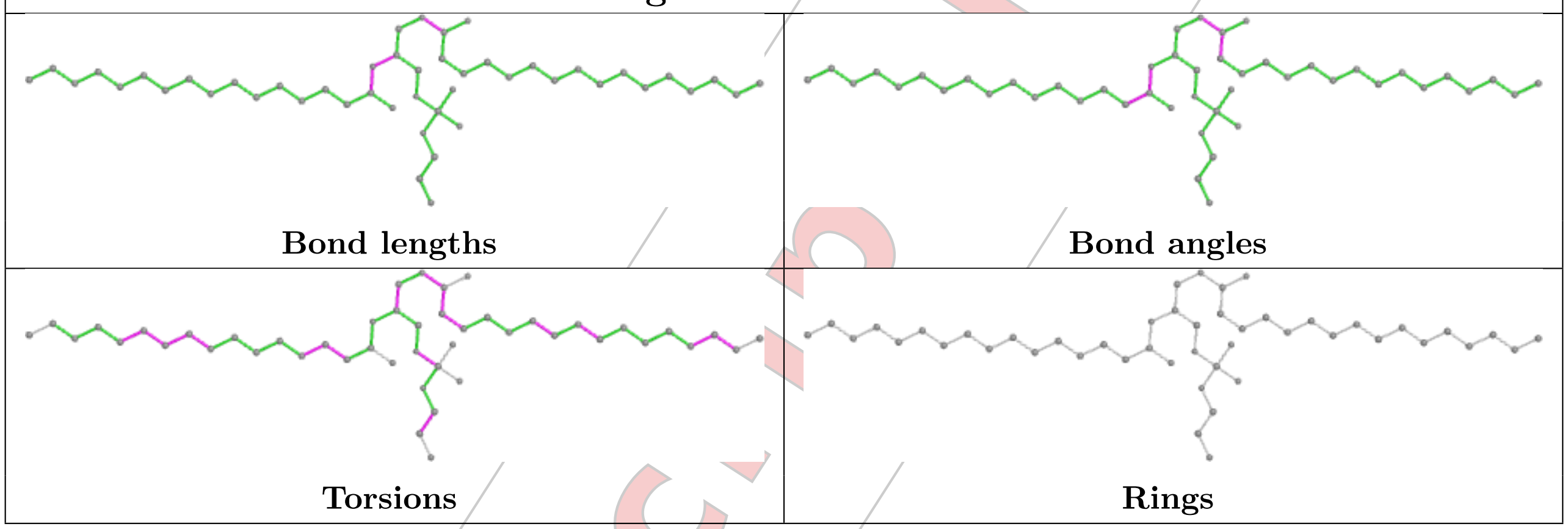

5.7 Other polymers (i)

There are no such residues in this entry.

5.8 Polymer linkage issues (i)

There are no chain breaks in this entry.

For Manuscript Review

6 Fit of model and data (i)

6.1 Protein, DNA and RNA chains (i)

In the following table, the column labelled '#RSRZ> 2' contains the number (and percentage) of RSRZ outliers, followed by percent RSRZ outliers for the chain as percentile scores relative to all X-ray entries and entries of similar resolution. The OWAB column contains the minimum, median, 95th percentile and maximum values of the occupancy-weighted average B-factor per residue. The column labelled 'Q< 0.9' lists the number of (and percentage) of residues with an average occupancy less than 0.9.

Mol	Chain	Analysed	<RSRZ>	#RSRZ>2			OWAB(Å^2)	Q<0.9
1	A	256/258 (99%)	-0.22	2 (0%)	82	79	44, 67, 100, 113	0
1	B	256/258 (99%)	-0.10	1 (0%)	89	86	44, 72, 108, 133	0
1	C	256/258 (99%)	-0.22	1 (0%)	89	86	49, 68, 95, 115	0
1	D	245/258 (94%)	0.54	19 (7%)	20	17	57, 99, 137, 166	0
1	E	258/258 (100%)	-0.23	3 (1%)	76	72	45, 67, 99, 123	0
1	F	255/258 (98%)	0.62	15 (5%)	29	24	67, 106, 152, 177	0
All	All	1526/1548 (98%)	0.06	41 (2%)	56	50	44, 76, 129, 177	0

All (41) RSRZ outliers are listed below:

Mol	Chain	Res	Type	RSRZ
1	D	578	TYR	6.0
1	F	445	ASP	5.8
1	D	362	GLU	4.9
1	F	447	TRP	4.6
1	E	349	MET	4.4
1	F	354	TYR	4.1
1	D	389	HIS	3.8
1	F	352	PRO	3.5
1	D	379	LEU	3.5
1	D	390	CYS	3.5
1	D	367	TYR	3.3
1	F	465	TYR	3.0
1	F	359	HIS	2.9
1	D	363	GLU	2.9
1	E	351	CYS	2.9
1	F	522	ILE	2.8
1	D	573	PHE	2.8
1	F	462	LEU	2.8
1	F	513	LEU	2.8

Continued on next page...

For Manuscript Review

Continued from previous page...

Mol	Chain	Res	Type	RSRZ
1	D	396	GLN	2.7
1	D	576	VAL	2.7
1	D	513	LEU	2.7
1	F	510	TRP	2.6
1	A	444	THR	2.6
1	D	382	SER	2.5
1	D	400	SER	2.5
1	D	487	ILE	2.4
1	E	575	LYS	2.4
1	A	354	TYR	2.3
1	D	582	GLU	2.2
1	D	371	LEU	2.2
1	F	526	PHE	2.1
1	D	366	ILE	2.1
1	F	531	TYR	2.1
1	B	555	LEU	2.1
1	D	386	ALA	2.1
1	F	463	ASN	2.1
1	C	485	THR	2.0
1	F	371	LEU	2.0
1	D	522	ILE	2.0
1	F	388	LEU	2.0

6.2 Non-standard residues in protein, DNA, RNA chains (i)

There are no non-standard protein/DNA/RNA residues in this entry.

6.3 Carbohydrates (i)

There are no monosaccharides in this entry.

6.4 Ligands (i)

In the following table, the Atoms column lists the number of modelled atoms in the group and the number defined in the chemical component dictionary. The B-factors column lists the minimum, median, 95th percentile and maximum values of B factors of atoms in the group. The column labelled 'Q< 0.9' lists the number of atoms with occupancy less than 0.9.

Mol	Type	Chain	Res	Atoms	RSCC	RSR	B-factors(Å^2)	Q<0.9
2	PEF	B	701	47/47	0.83	0.22	47,77,111,134	0

Continued on next page...

For Manuscript Review

Continued from previous page...

Mol	Type	Chain	Res	Atoms	RSCC	RSR	B-factors(Å^2)	Q<0.9
2	PEF	A	701	47/47	0.85	0.21	53,77,114,132	0
2	PEF	D	701	47/47	0.85	0.23	70,97,151,168	0
2	PEF	C	701	47/47	0.86	0.20	45,75,123,132	0
2	PEF	E	701	47/47	0.86	0.20	51,72,100,128	0

The following is a graphical depiction of the model fit to experimental electron density of all instances of the Ligand of Interest. In addition, ligands with molecular weight > 250 and outliers as shown on the geometry validation Tables will also be included. Each fit is shown from different orientation to approximate a three-dimensional view.

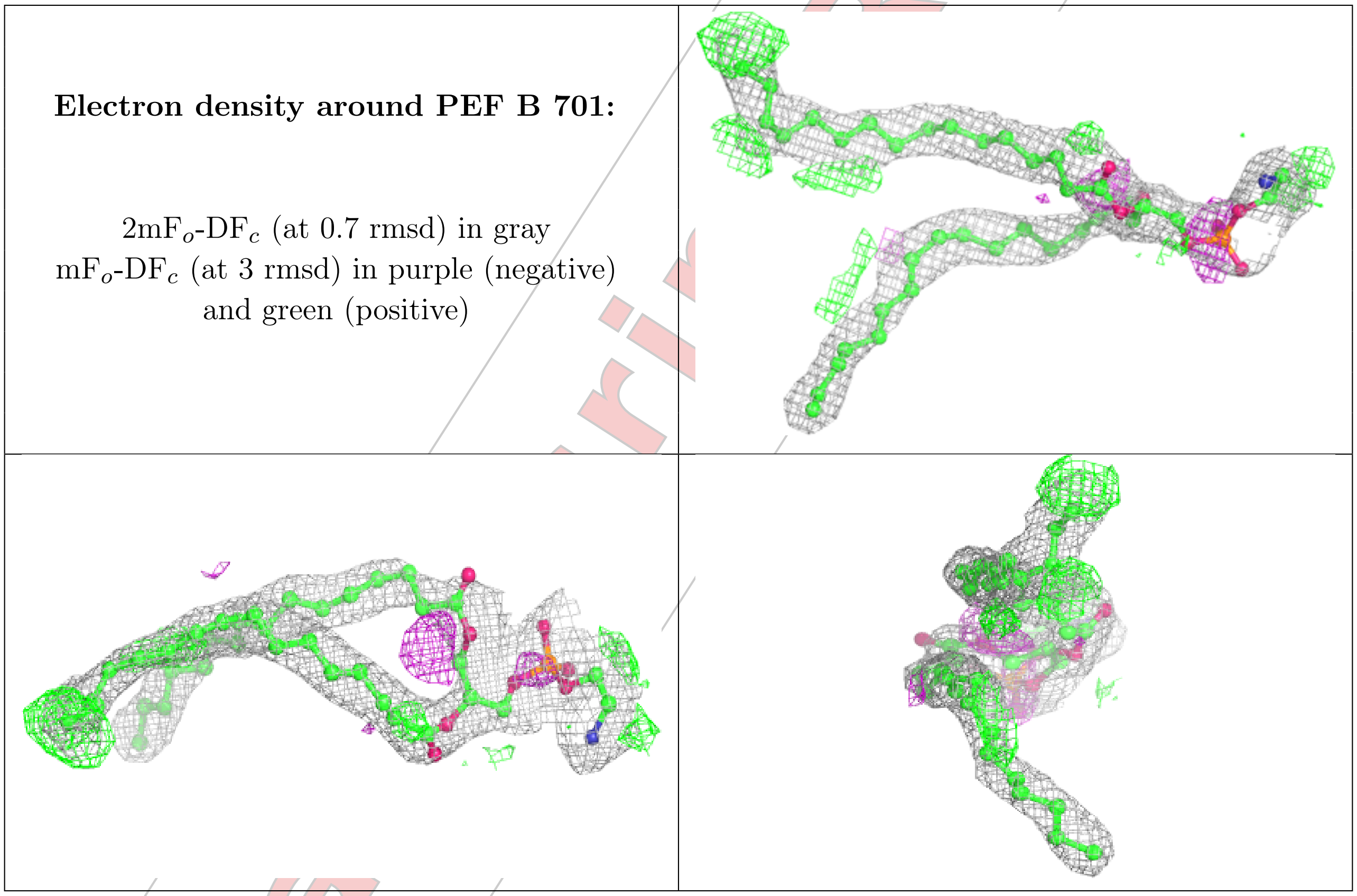

For Manuscript Review

Electron density around PEF A 701:

$2mF_o$-DF_c (at 0.7 rmsd) in gray
mF_o-DF_c (at 3 rmsd) in purple (negative)
and green (positive)

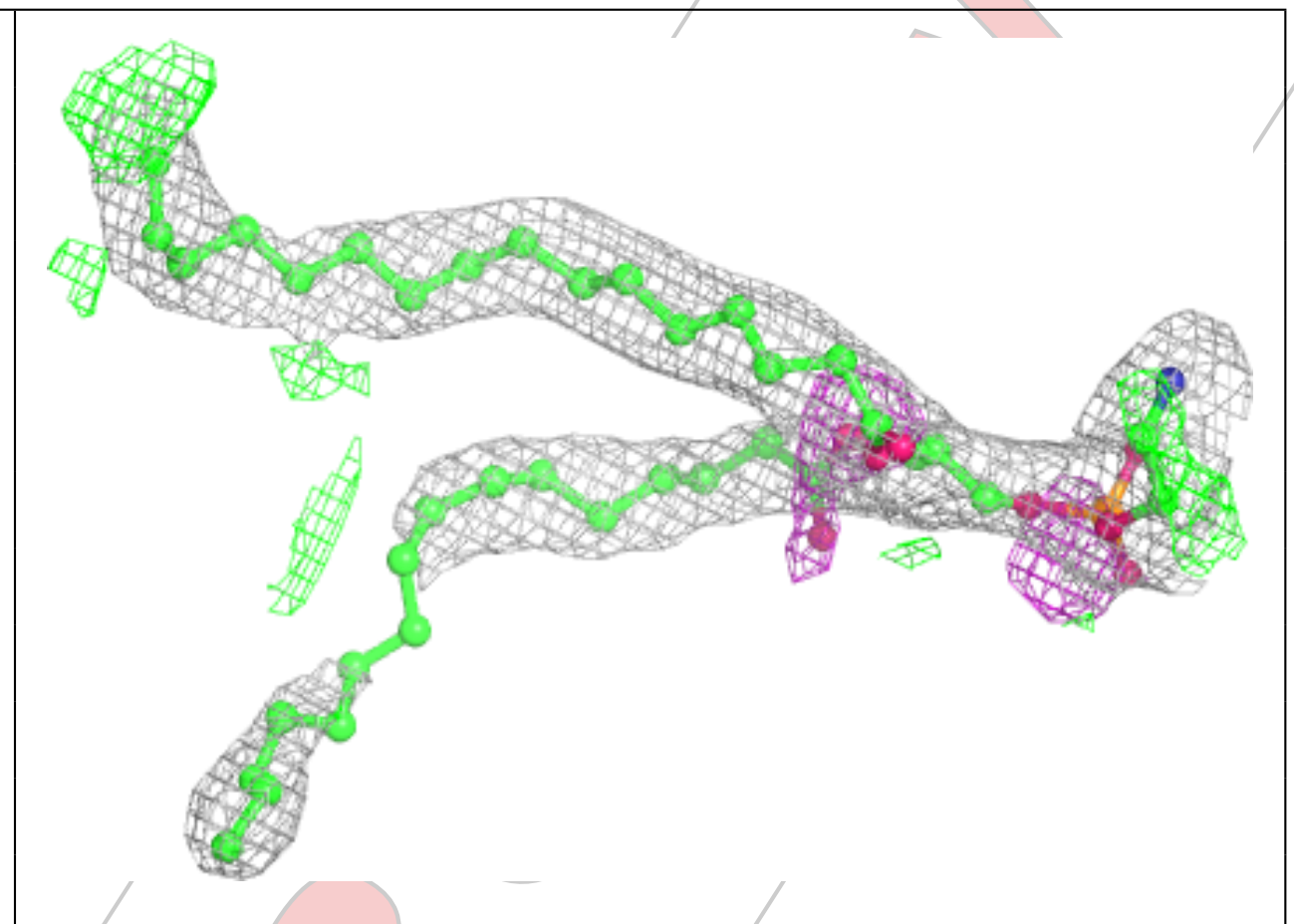

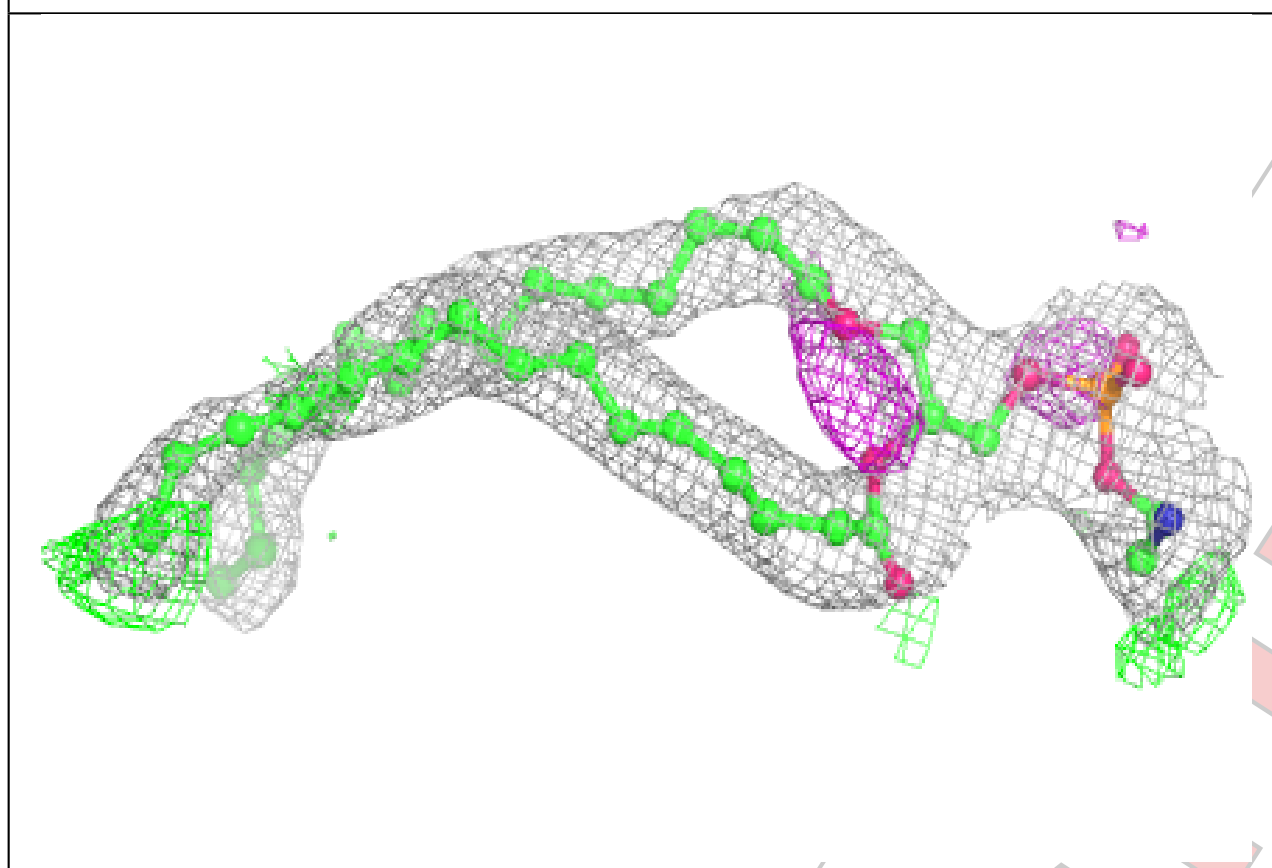

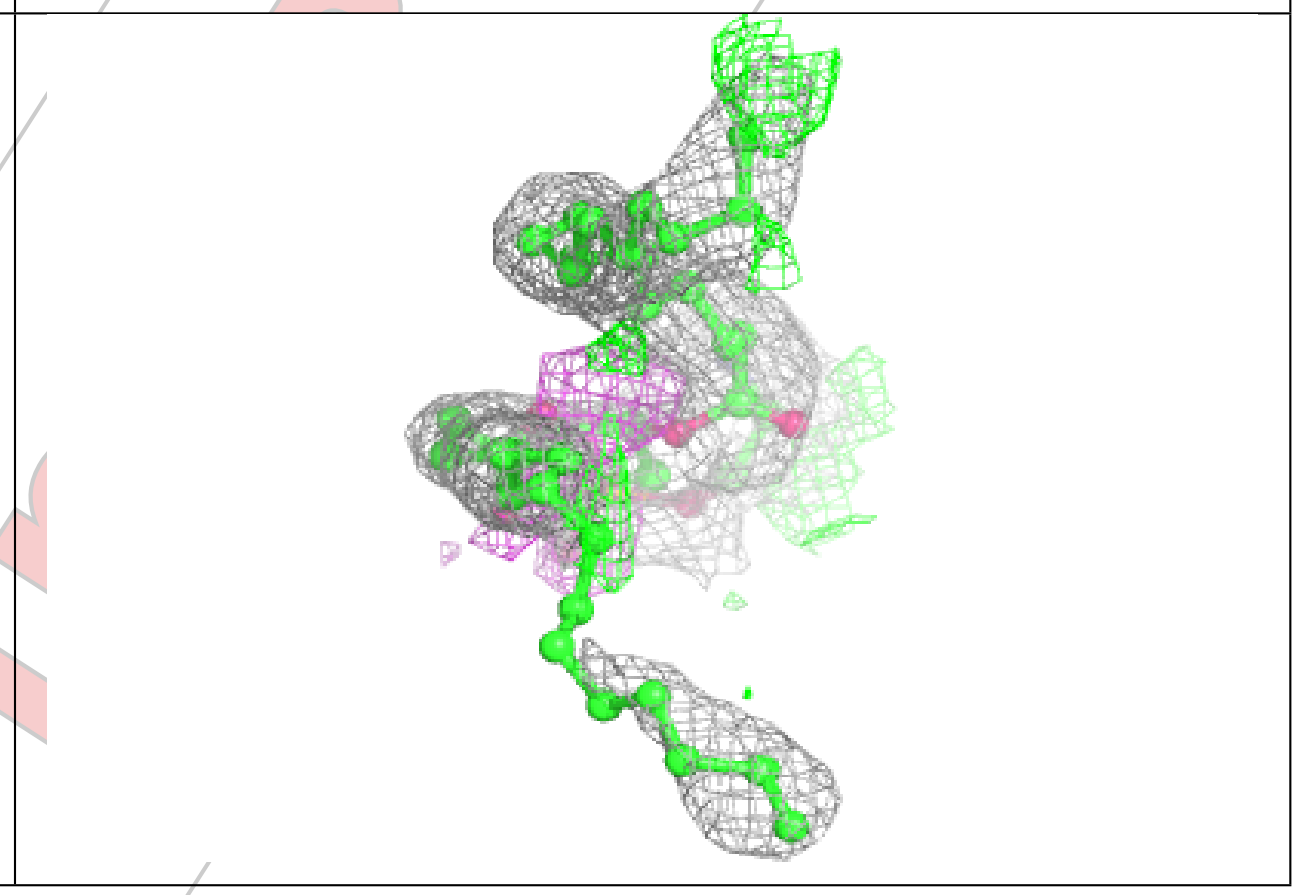

Electron density around PEF D 701:

$2mF_o$-DF_c (at 0.7 rmsd) in gray
mF_o-DF_c (at 3 rmsd) in purple (negative)
and green (positive)

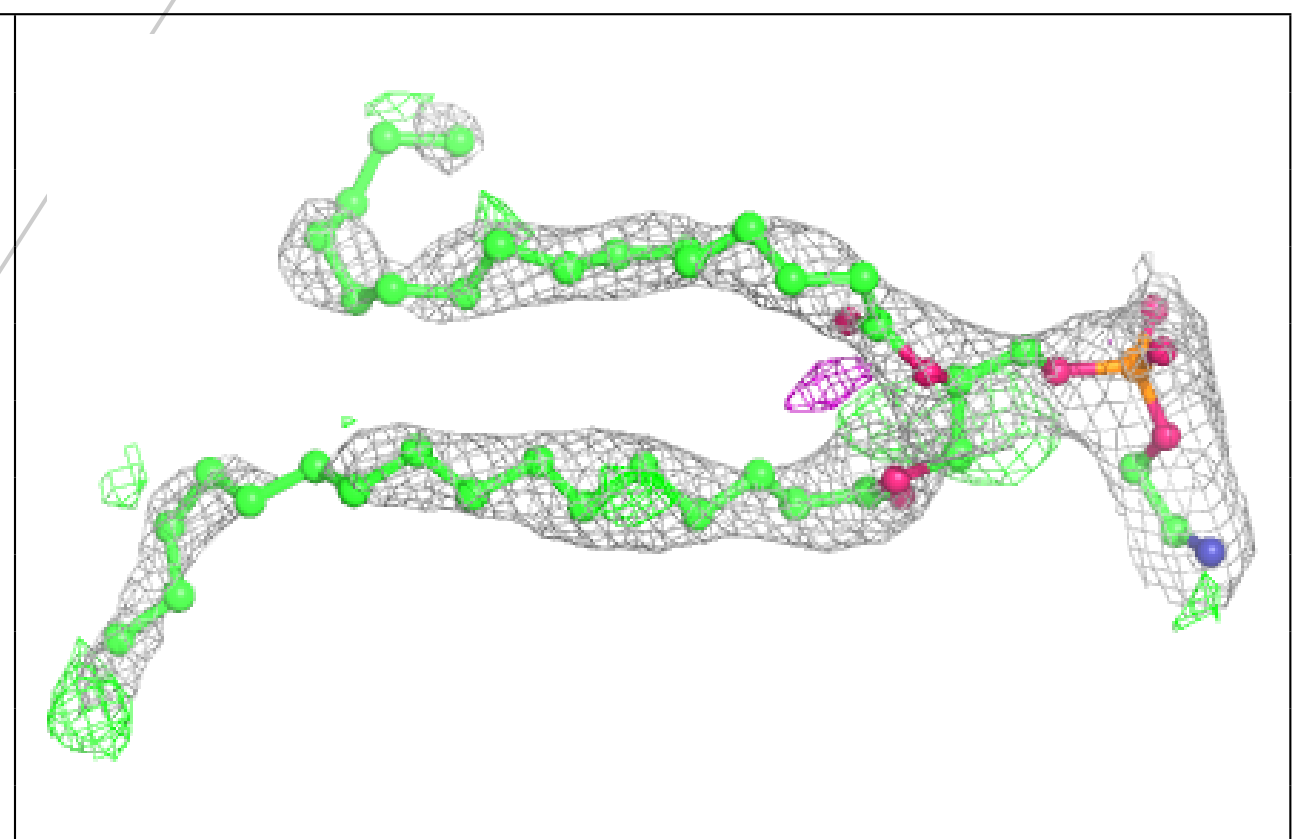

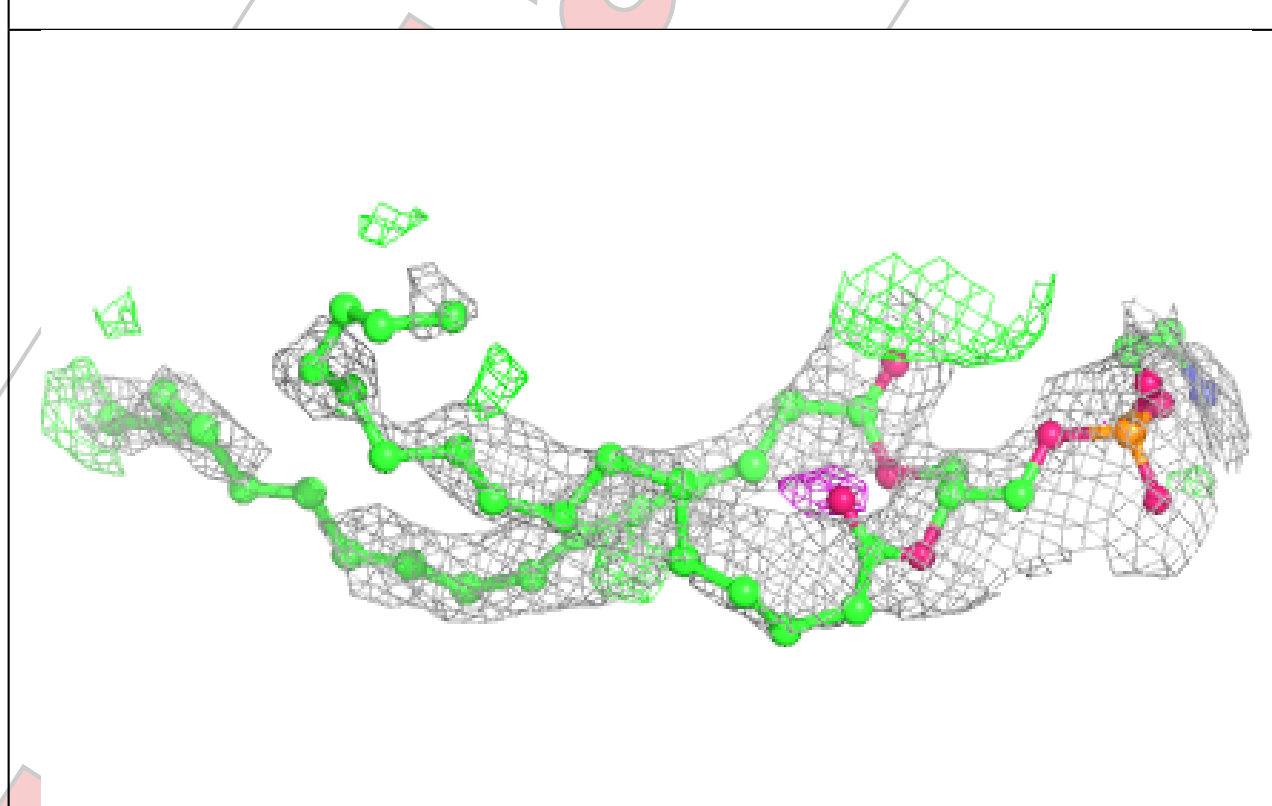

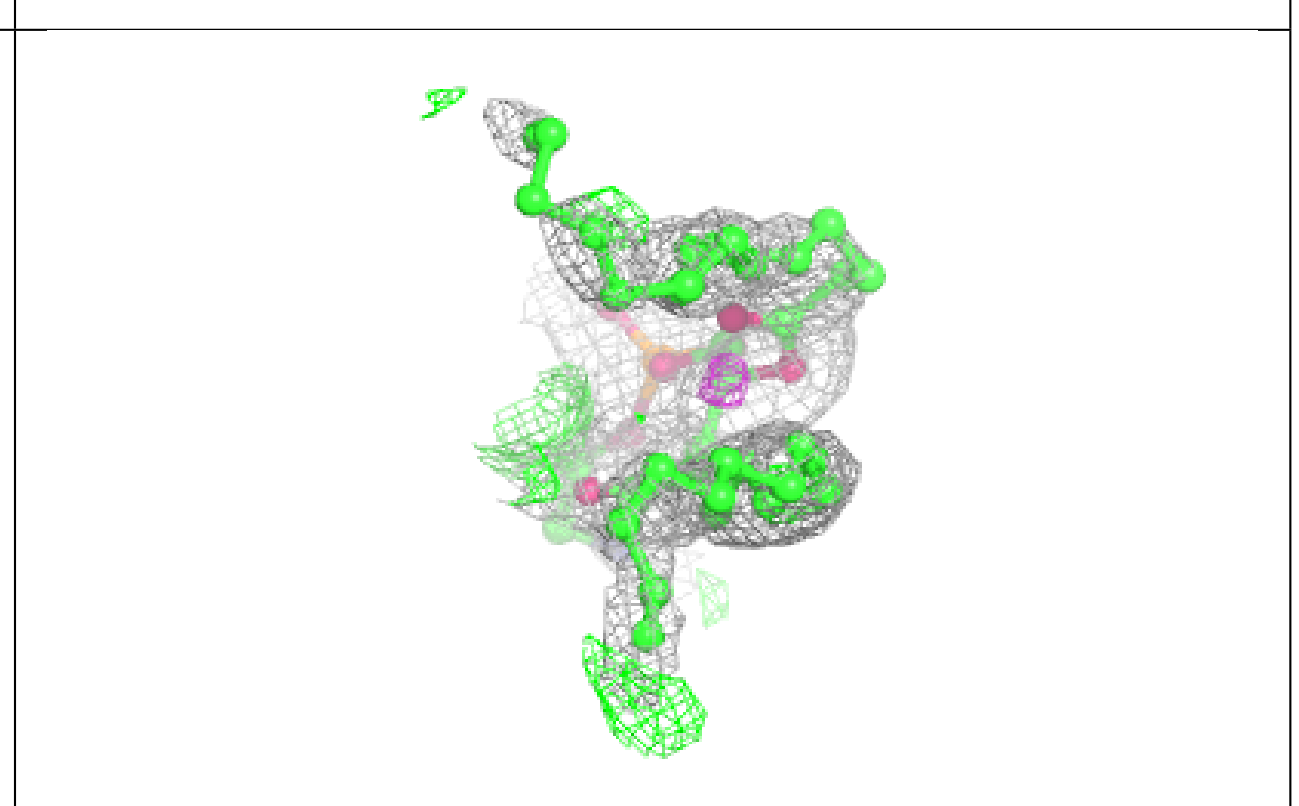

Electron density around PEF C 701:

$2mF_o$-DF_c (at 0.7 rmsd) in gray
mF_o-DF_c (at 3 rmsd) in purple (negative)
and green (positive)

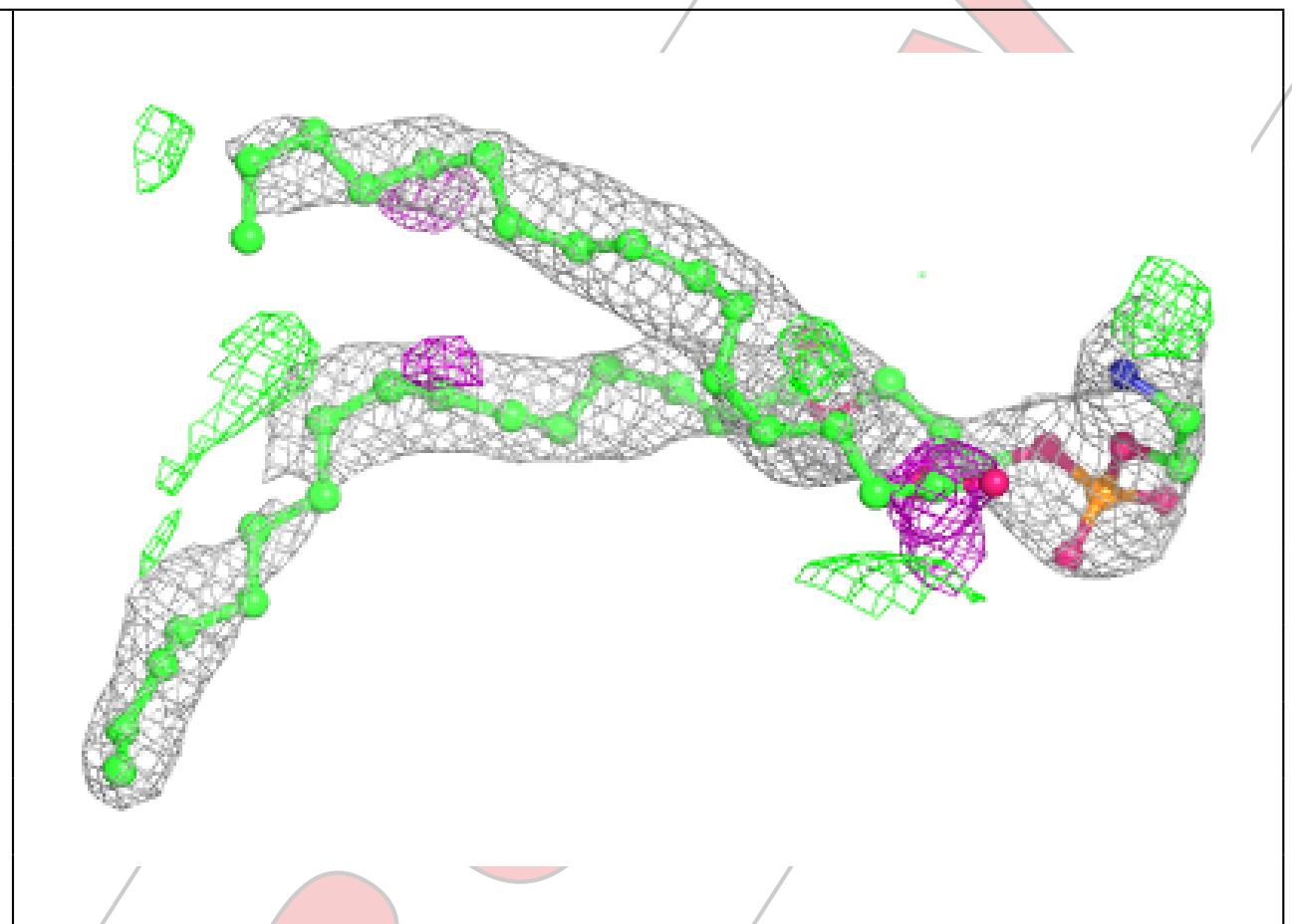

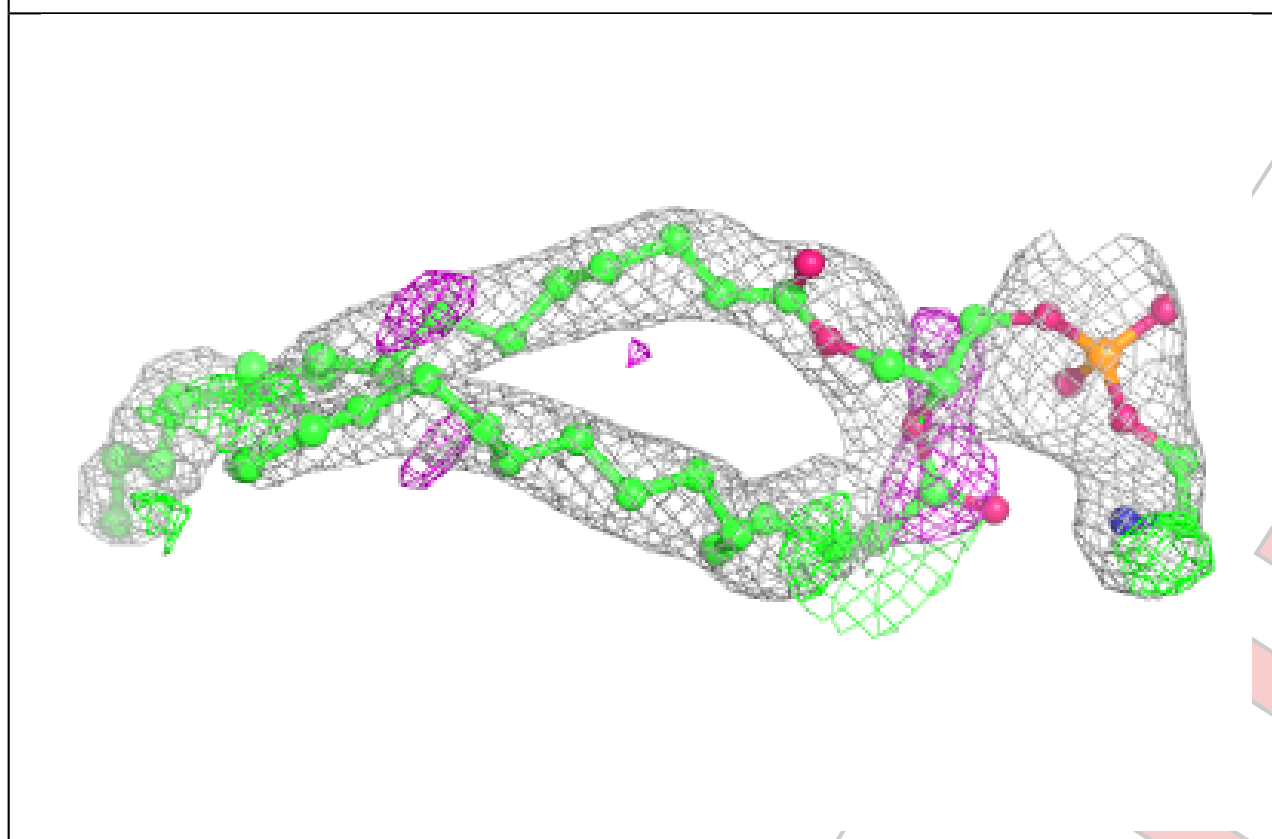

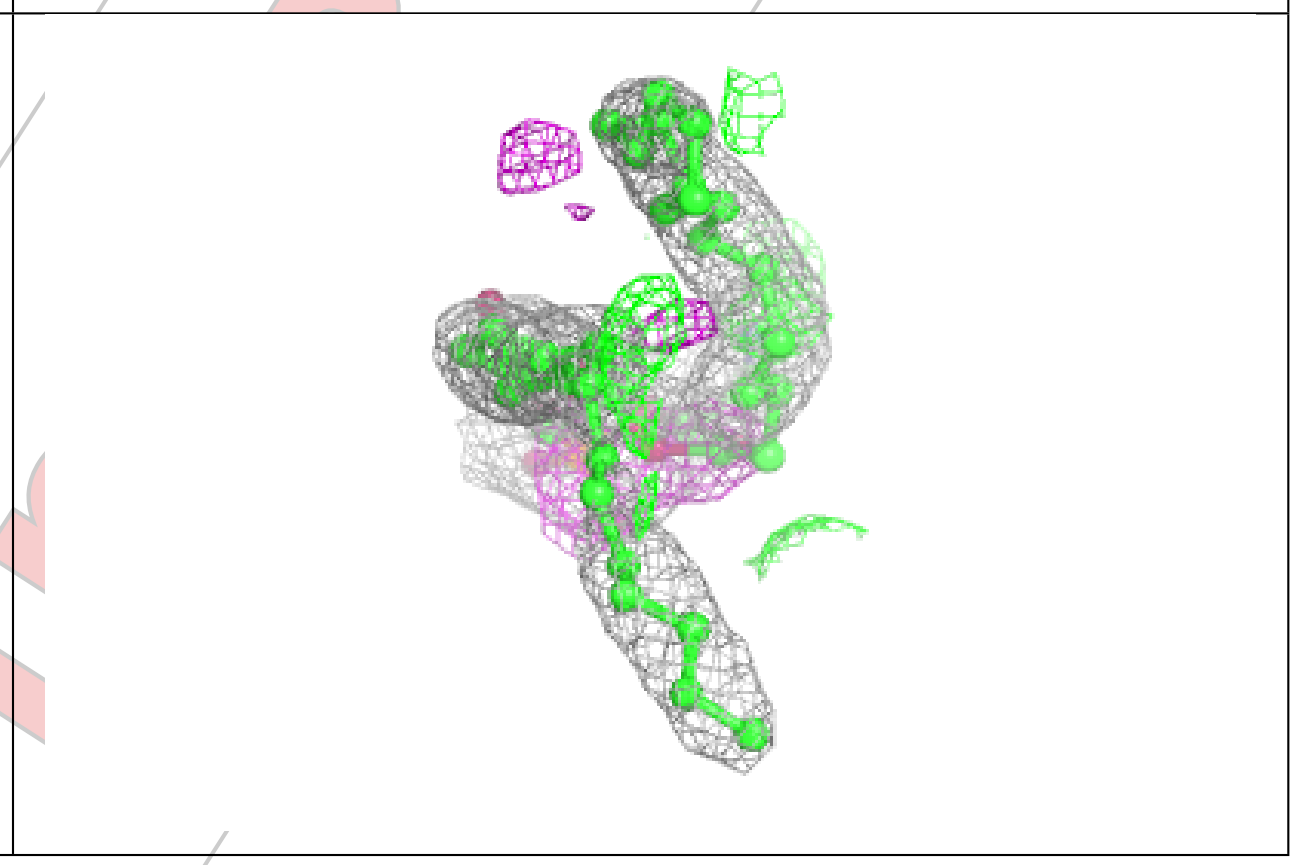

Electron density around PEF E 701:

$2mF_o$-DF_c (at 0.7 rmsd) in gray
mF_o-DF_c (at 3 rmsd) in purple (negative)
and green (positive)

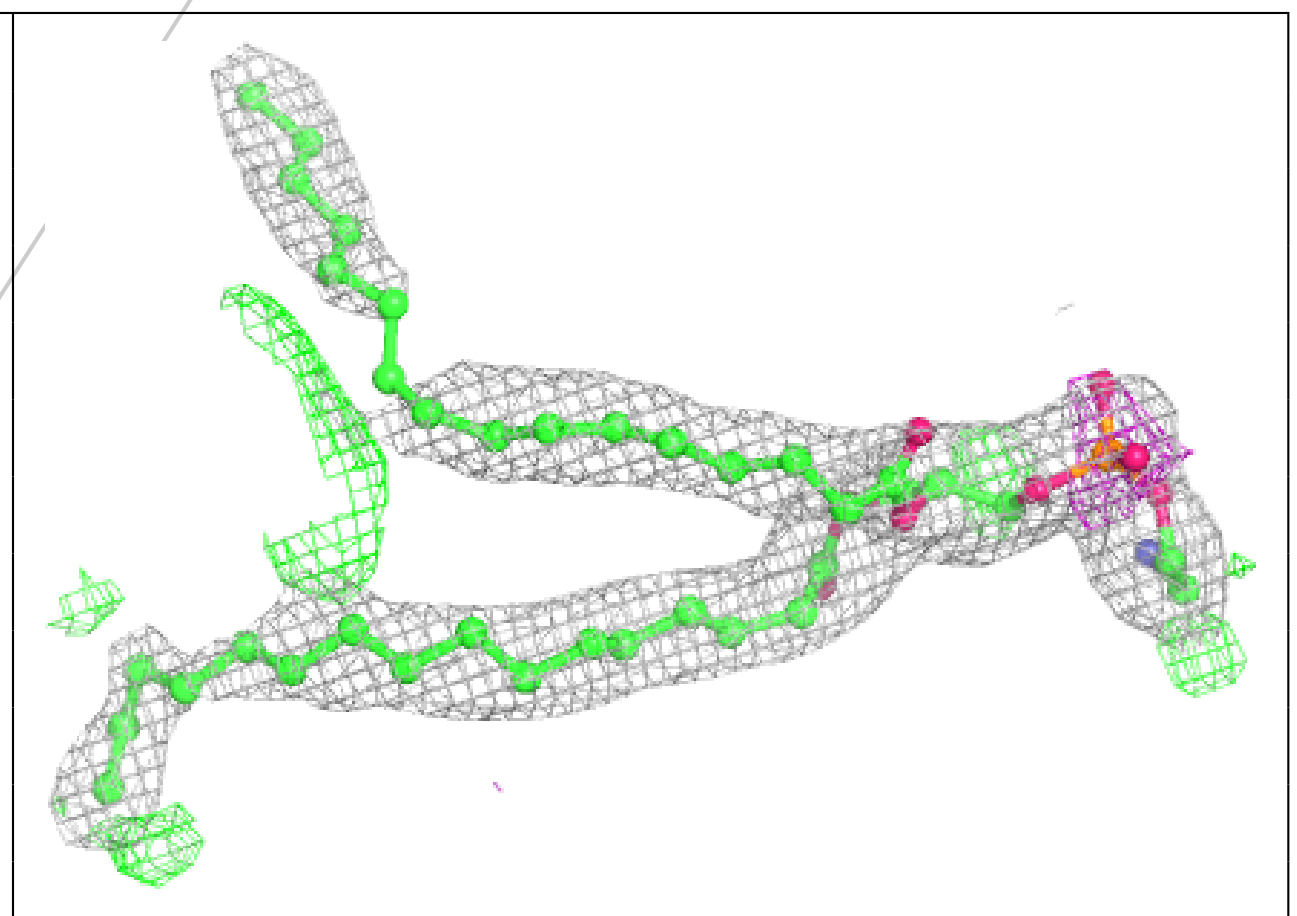

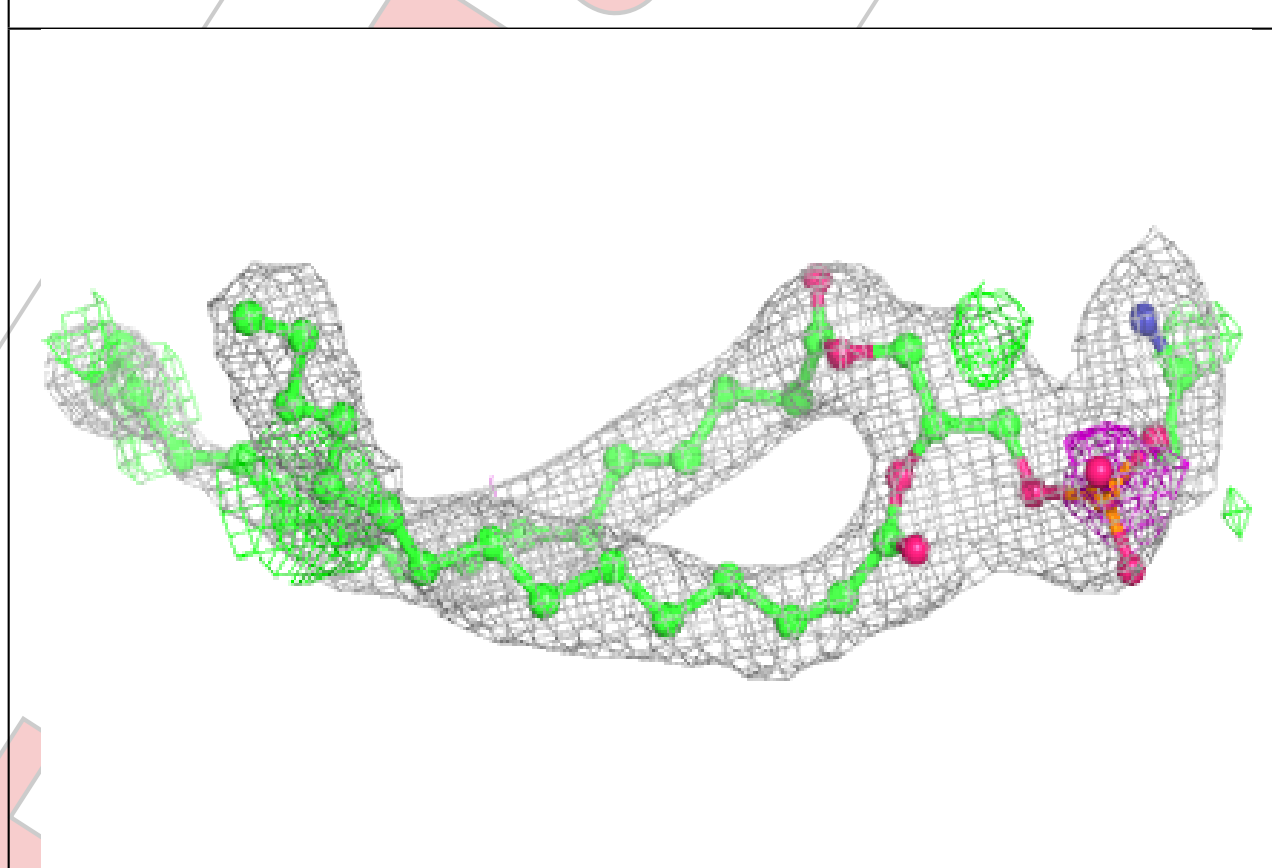

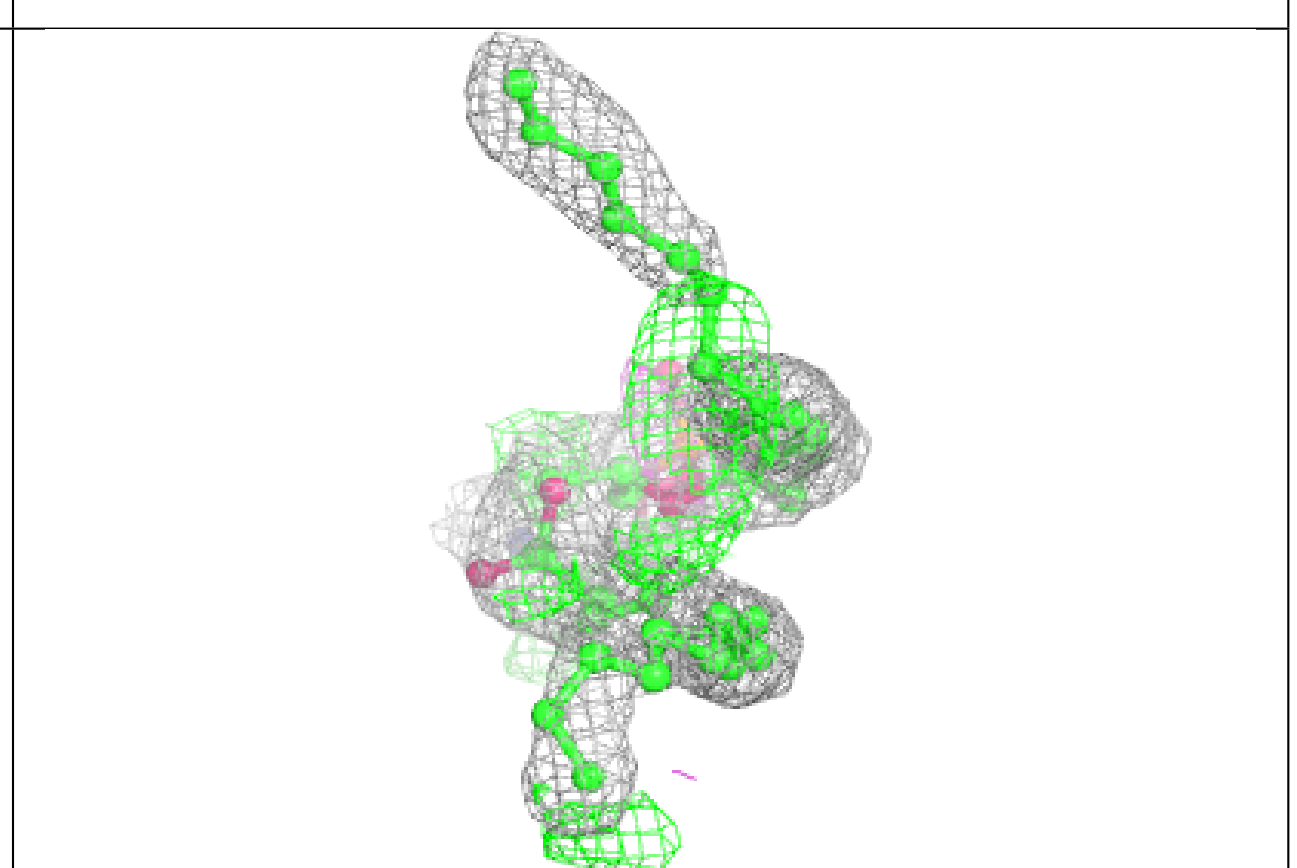

6.5 Other polymers (i)

There are no such residues in this entry.

For Manuscript Review
